# Antinoceptive and Anti-inflammatory Activities of the Ethanolic Extract, Fractions and Flavones Isolated from *Mimosa tenuiflora* (Willd.) Poir (Leguminosae)

**DOI:** 10.1371/journal.pone.0150839

**Published:** 2016-03-08

**Authors:** Mariluze P. Cruz, Cassya M. F. Andrade, Kelle O. Silva, Erika P. de Souza, Regiane Yatsuda, Lucas M. Marques, Juceni P. David, Jorge M. David, Marcelo H. Napimoga, Juliana T. Clemente-Napimoga

**Affiliations:** 1 Instituto Multidisciplinar em Saúde, Universidade Federal da Bahia, Vitória da Conquista, BA, Brazil; 2 Faculdade de Farmácia, Universidade Federal da Bahia, Salvador, BA, Brazil; 3 Instituto de Química, Universidade Federal da Bahia, Salvador, BA, Brazil; 4 Laboratory of Immunology and Molecular Biology, São Leopoldo Mandic Institute and Research Center, Campinas, SP, Brazil; 5 Instituto de Biologia, Universidade Estadual de Campinas, Campinas, SP, Brazil; University of British Columbia, CANADA

## Abstract

The bark of *Mimosa tenuiflora* (Willd.) Poiret (Leguminosae family), popularly known as “jurema preta” in Brazil, is used by the population of Contendas of Sincorá (Bahia State, Brazil) for the treatment of coughs and wound healing. Thus, the aim of this study was to evaluate the antinociceptive and anti-inflammatory activities of the bark ethanol extract (EEMT) and solvent soluble fractions (hexane—H, DCM—D, EtOAc—E and BuOH—B) of the extract *in vivo*. Additionally, we synthesized 5,7-dihidroxy-4’-methoxyflavanone (isosakuranetin) and isolated the compound sakuranetin, and both compounds were also tested. The anti-inflammatory and antinociceptive assays performed were: writhing test; nociception induced by intraplantar formalin injection; leukocyte recruitment to the peritoneal cavity; evaluation of vascular permeability (Evans blue test); and evaluation of mechanical hypernociception (von Frey test). Production of TNF-α, IL-10, myeloperoxidase and the expression of ICAM-1 were also evaluated. Statistical analysis was performed by one-way ANOVA followed by the Bonferroni post-test (n = 8), with *P* < 0.05. The EEMT showed antinociceptive activities in writhing test (100–200 mg/kg), in the second phase of the formalin test (50–200 mg/kg), and in mechanical hypernociception (100 mg/kg). EEMT showed an anti-inflammatory effect by reducing neutrophil migration to the peritoneal cavity and in the plantar tissue detected by the reduction of myeloperoxidase activity (100 mg/kg), reduction of IL-10 levels and expression of ICAM-1 in the peritoneal exudate and the mesentery (100 mg/kg), respectively. The four soluble EEMT fractions showed good results in tests for antinociceptive (H, D, E, B) and anti-inflammation (H, D, E). Only sakuranetin showed reduction of the writhing and neutrophil migration (200 mg/kg). Thus, the EEMT and soluble fractions of *M*. *tenuiflora* bark demonstrated great antinociceptive and anti-inflammatory activities, as also sakuranetin. More studies should be conducted to elucidate the mechanism of action of this compound. To the best of our knowledge, this is the first report on the antinociceptive activity of the *M*. *tenuiflora* fractions and the bioactive isolated compound sakuranetin *in vivo*.

## Introduction

Inflammation is characterized by pain, redness, swelling and dysfunction of the tissues and organs and is the normal result of host protective responses to tissue injury caused by numerous stimuli (e.g., physical trauma, chemicals and infectious agents) [1,2]. Inflammation is a hallmark of many chronic diseases; however, the long-term use of steroids and/or non-steroid anti-inflammatory drugs leads to hormonal side effects and gastric lesions in many instances [3,4]. Inflammation is commonly associated with pain as a secondary process due to the secretion of algesic mediators [5].

Pain represents the primary symptom for the diagnosis of several disease conditions and is widely accepted as one of the most important determinants of quality of life. Currently, most conventional pain therapies are unsatisfactory in terms of efficacy, tolerability and toxicity [6]. Despite recent progress in the development of therapy, there is still a need for effective and potent analgesics and anti-inflammatories, especially for the treatment of chronic pain.

Natural products have contributed enormously to the development of important therapeutic drugs currently used in modern medicine [7–9]. There is increasing recognition that natural products may provide a viable source for new drug molecules, especially in view of the failure of the more popular combinatorial conventional synthetic chemistry approaches [10].

**Fig 1:**
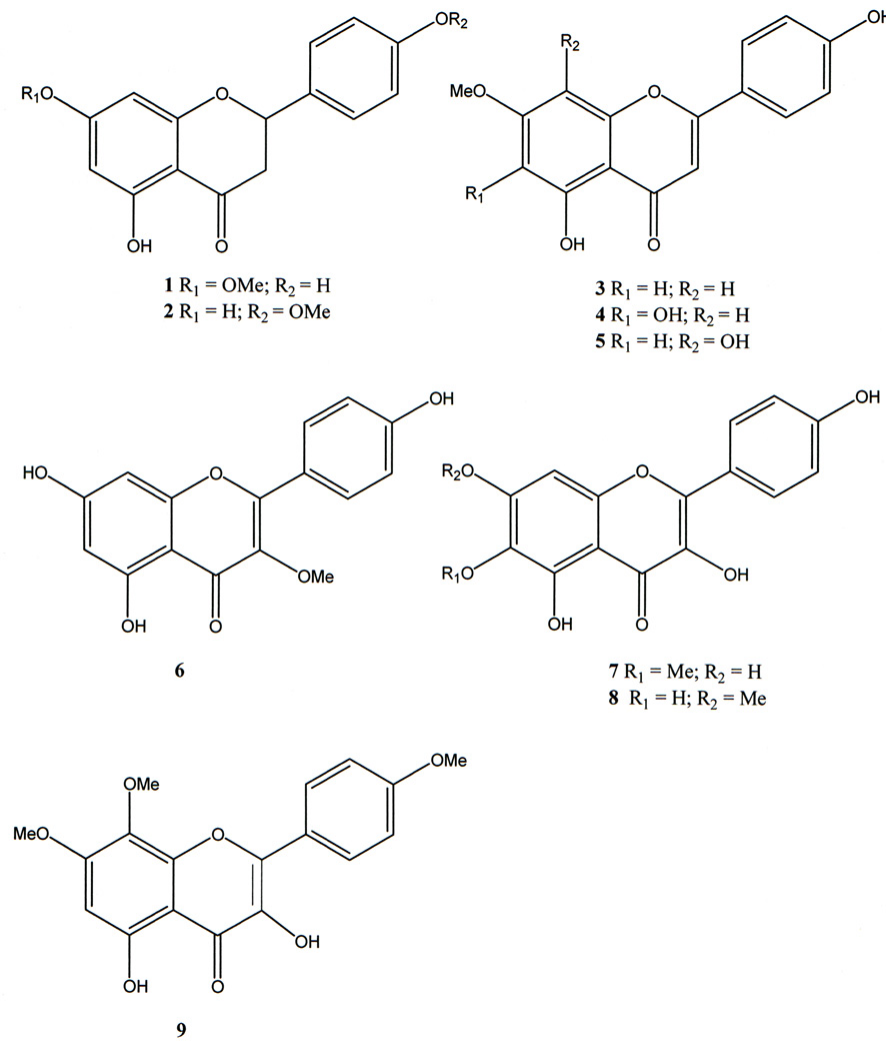
Flavonoids from *Mimosa tenuiflora*.

*Mimosa tenuiflora* (Willd.) Poiret (Fig 1) (also known as *Mimosa hostilis* (Mart.) Benth and *Acacia hostilis* Benth.) is a plant of the Leguminosae family and is popularly known as “calumbi” and “jurema preta” in Brazil [11,12]. *M*. *tenuiflora* is a common shrub/tree that is distributed in areas of tropical deciduous forests in the Americas from the southeastern regions of Mexico to northern Brazil and Venezuela, where it grows as secondary opportunistic vegetation [13]. It is a legume tree 5 to 7 m tall and has a high level of tannins and it is an important forage plant used to feed small ruminants in the “caatinga” (a semi-desertic vegetation in interior of Brazilian northeastern) during the dry season [14].

In northeastern Brazil, some indigenous tribes make use of the bark and roots for psychoactive purposes in religion and medicine [15]. The substance responsible for its psychoactivity is a tryptaminic alkaloid called *N*,*N*-dimetyltryptamine (DMT). Additionally, alkaloids, labdane diterpenoids and flavonoids were previously isolated from *M*. *hostilis* [16].

Other studies of *M*. *tenuiflora* showed potential cicatrizing properties *in vitro* [17–19]. According to Mexican ethnobotanic sources, the direct application of the dried powdered bark to the lesion was an effective remedy for treating skin burns and wounds [20,21]. The population of the district of Palmeiras at Contendas of Sincorá (Bahia State, Brazil) uses the bark of *M*. *tenuiflora* for the treatment of coughs and wound healing [22]. A standardized tannin content extract obtained from the *M*. *tenuiflora* bark also showed excellent therapeutic properties for the treatment of skin venous leg ulcerations and strong *in vitro* antimicrobial properties against a wide group of microorganisms [23].

Although the chemical composition of these plants has been investigated, few studies have investigated the pharmacological properties of the newly identified compounds. This paper describes the isolation of 5,4’-dihydroxy-7-methoxyflavanone (sakuranetin), 5,4’-dihydroxy-7-methoxyflavone (genkwanin), 5,6,4’-trihydroxy-7-methoxyflavone (sorbifolin), 5,4’-di-hydroxy-7,8-dimethoxyflavone, and 5,7,4’-trihydroxy-3-methoxyflavone and the minor flavonols 5,7,4’-trihydroxy-6-methoxyflavonol, 5,6-dihydroxy-7,4’-dimethoxyflavonol and 5-hydroxy-7,8,4’-trimethoxyflavonol from the leaves of *M*. *tenuiflora*.

In previous studies dealing with this plant, isosakuranetin (5,4’-dihydroxy-7-methoxyflavanone) was identified as the main flavone present in the extract; however, the literature NMR data for these two isomers are confusing. To unequivocally determine the structure of this *Mimosa* flavone, isosakuranetin was synthetized from fluoroglucinol and 4-methoxybenzaldheyde. The structures of all compounds were determined based on spectrometric data (NMR, MS, UV and IR). These procedures confirmed that sakuranetin was the main flavone present in *M*. *tenuiflora*.

Thus, the antinociceptive and anti-inflammatory activities of the bark ethanol extract, solvent soluble fractions, isosakuranetin and sakuranetin were evaluated *in vivo*. To the best of our knowledge, this is the first report on the *in vivo* antinociceptive activity of the fractions and the isolated compound sakuranetin of *M*. *tenuiflora*.

## Materials and Methods

### Drugs section

Carrageenan (Cg), acetic acid, ethanol (EtOH), hexane, dichloromethane (DCM), ethyl acetate (EtOAc), butan-1-ol (BuOH) and formaldehyde were purchased from Sigma (St. Louis, MO, USA). Indomethacin was purchased from Merck Sharp & Dohme (São Paulo, SP, Brazil), Tumor necrosis factor- α (TNF-α) and interleukin-1β (IL-1β) were purchased from R&D Systems (Pittsburgh, PA, USA). Ketamine and xylazine were obtained from Syntec Laboratory (Cotia, SP, Brazil). Morphine was obtained from Cristalia (Itapira, SP, Brazil).

### General procedures

The ^1^H (300 MHz), ^13^CNMR and DEPT (75 MHz), ^1^H-^1^H COSY and ^1^H-^13^C COSY experiments were performed in a Varian mod. Gemini 2000. HMQC and HMBC were run in a Bruker DRX 500; chemical shifts were recorded in δ (ppm) from the solvent peak relative to the tetramethylsilane (TMS).The UV spectra were analyzed in a Varian CARY I spectrophotometer. The infrared spectra were recorded in a Shimadzu (mod. Iraffinity-1) spectrophotometer. Optical rotations were measured in a Perkin Elmer Polarimeter (mod. 341). The GC-MS analyses were performed in a QP2010SE Shimadzu mass spectrometer (mod. GC2010 Plus) employing an Rtx-5MS (30 m, 0.25 mm diameter, film thickness 0.25 μm) column Prior to injection, the samples (3 mg) were dissolved in 60 μL of pyridine in capped vials, followed by the addition of 100 μL of *N*,O-bis(trimethylsilyl)trifluoroacetamide (BSTFA) containing 1% trimethylchlorosilane (TMCS) (Sigma-Aldrich®) for derivatization. The mixture was heated at 70°C for 30 min, and a 1 μL of mixture was injected in the GC-MS [24]. The GC conditions were a temperature of 290°C in the injector, initial temperature of 80°C in the oven for 5 min and a final temperature of 285°C (range 4°C/min). The conventional chromatographic methods used the CC silica gel 60 (63–200 μm) from Acros, Sephadex LH-20 (Sigma) and precoated Silica gel TLC plates (Whatman) to monitor the chromatographic fractions that were revealed by employing iodine fumes and UV light (254/366 nm).

### Plant material

The plant was collected (IBAMA’s license # N 12292–1) in the National Forest Contendas do Sincorá (S13°55'23.2'' and W041°07'05.0'', at 374 m height) situated in the municipality of Contendas do Sincorá, Bahia State, Northeast Brazil in august of 2009. The plant material was identified by Prof. Avaldo de Oliveira S. Filho, and voucher specimens (n° HUESBVC 4907, 4980 and 4993) were deposited in the Herbarium of Universidade Estadual do Sudoeste da Bahia (UESB) in Vitória da Conquista, Bahia, Brazil.

### Extraction and isolation of flavonoids

The dry bark (301 g), leaves (200 g) and branches (420 g) were subjected to extraction with EtOH 3 times, resulting in 49.7 g (bark), 41.2 g (leaves) and 31.9 g (branches) ethanolic extracts. The extracts were sequentially dissolved in a mixture of EtOH:H_2_O (1:1) and partitioned between hexane, DCM, EtOAc and BuOH (Table 1).

**Table 1:** Mass quantities of the fractions obtained from the partitioned ethanolic extract of *Mimosa tenuiflora*.

Fractions	Barks (32 g)	Leaves (37 g)	Branches (20 g)
Hexane	0.78 g	11.30 g	0.90 g
DCM	0.76 g	7.85 g	1.10 g
EtOAc	15.10 g	2.48 g	3.18 g
BuOH	8.78 g	4.32 g	3.69 g

The DCM soluble fraction of the EtOH extract of the leaves (18.8 g) was subjected to a CC silica gel and eluted with mixtures of CHCl_3_:EtOAc to obtain 17 fractions. The fraction eluted with CHCl_3_:EtOAc (4:1) was chromatographed in a Sephadex LH-20 and eluted with DCM:MeOH (1:1), which permitted the isolation of 162.3 mg of compound **1** (sakuranetin) and compound **3** (21 mg). The fraction eluted from CC with CHCl_3_:EtOAc (3:2) was rechromatographed employing a CC silica gel and hexane:EtOAc (4:1), which permitted the isolation of isolate **5** (18 mg). The fraction eluted with CHCl_3_:EtOAc (1:1) was subjected to a Sephadex LH-20 CC and permitted the isolation of isolate **9** (5 mg). A mixture of flavonoids were isolated in another Sephadex LH-20 and eluted with a mixture of (CH_3_)_2_CO:MeOH (1:1), permitting the isolation of isolates **7** (7 mg), **8** (10 mg) and **4** (45.2 mg). Compound **6** (10 mg) was isolated by preparative TLC eluted with DCM:MeOH (92:7).

### Synthesis of isosakuranetin

A solution of phloroglucinol (1) (Merck, 2.5 g, 198 mmol) and acetic anhydride (Merck, 19 mL, 199.5 mmol) in a BF_3_OEt_2_ solution (Quell, 7.5 mL) were stirred at room temperature for 34 h (S2 Fig). A solution of 10% sodium acetate (NaOAc) (Modern Chemistry, 7 mL) was added to this mixture and left to stand overnight. The precipitate was filtered, washed with water and dried to obtain 2.4 g of 2,4,6-hydroxyacetophenone (2) [25]. After confirming the product identity by the NMR and IR spectra, this procedure was repeated to obtain a greater amount of 2,4,6-hydroxyacetophenone.

Dry potassium carbonate (K_2_CO_3_, 828 mg, 6 mmol) and benzyl bromide (1.026 g, 800 μL, 6 mmol, Aldrich®) were added to a solution of 504 mg of 2,4,6-tri-hydroxyacetophenone dissolved in 4 mL of hexamethylphosphoramide (HMPA, Sigma-Aldrich®). The mixture was subjected to magnetic stirring for 6 days; then, the reaction mixture was poured into 20 mL of water and extracted with ether (1 x 10 mL, 4 x 5 mL). The organic layer was washed with distilled water (3 x 5 mL), followed by a solution of 5% KOH (3 x 5 mL), distilled water again (3 x 5 mL) and 2 x 5 mL of brine (3.5% of NaCl solution). The solution was dried with MgSO_4_ and evaporated to obtain 428 mg (84.9%) of 4,6-dibenzyloxy-2-hydroxyacetophenone as a pale yellow oil [26]. The product was identified, and this procedure was repeated to obtain a greater mass of 4.

Anisaldehyde (60 μL, 68 mg, 1.46 mmol, Merck) was added to 96 mg of 4,6-dibenzyloxy-2-hydroxyacetophenone (4) dissolved in 10 mL of methanol in a round bottom flask. The mixture was mildly heated to obtain a homogeneous solution. After cooling, sodium methoxide (162 mg, 0.5 mL, 8.97 mmol) was added, and the mixture was stirred at room temperature for 11 days. The yellow suspension was transferred to a separatory funnel containing 30 mL of water and 50 mL of EtOAc (~ 50°C). The phases were separated, and the organic phase was washed with distilled water (3 x 15 mL) and brine (1 x 30 mL). Then, the solution was dried over MgSO_4_ and evaporated to obtain 124.2 mg (93.2%) of 2',4'-dibenzyloxy-6'-hydroxy-4-metoxichalcone (6) as a yellow solid. After spectral identification, this procedure was repeated to obtain a higher amount of this chalcone.

Isosakuranetin was obtained from 90 mg of 2',4'-dibenzyloxy-6'-hydroxy-4-metoxichalcone dissolved in 8 mL of acetic acid under heating (90°C). After cooling to 60°C, 0.4 mL of aqueous HBr (48%, Vetec) was added. The solution turned red and was stirred and maintained at 60°C for 20 h. The mixture was poured into 50 mL of distilled water and then extracted with EtOAc (1 x 30 mL). The aqueous solution was washed with water (2 x 30 mL) and 5% NaHCO_3_ (2 x 30 mL), distilled water (1 x 30 mL) and brine (1 x 30 mL). After drying over MgSO_4_, the solvent was evaporated to obtain a crystal. The crystal was dissolved in EtOAc and fractionated on 60 CC silica gel with DCM as the eluent by gradually increasing the polarity with EtOAc. A total of 54.34 mg of isosakuranetin (60.4%) was obtained. These procedures permitted us to obtain isosakuranetin (compound **2)** in 4 steps with an overall yield of 7.9%.

### Determination of antinociceptive and anti-inflammatory activities

#### Animals

All experiments were performed with male *Balb/C* mice (20–25 g) obtained and maintained at our Animal Facility at the Multidisciplinary Health Institute of the Federal University of Bahia. The animals were maintained in a temperature-controlled room (22 ± 2°C) with controlled humidity (50–70%) and a 12 h light/dark cycle. The animals were kept in polypropylene boxes containing wood shavings at the base of the box with free access to food (Labina®, Purina) and filtered water. The mice were evenly distributed among the groups. All animals were allowed to acclimatize to the air-conditioned laboratory for at least two h before the tests, which were performed during the light cycle phase. Animal care and research protocols were in accordance with the principles and guidelines adopted by the Brazilian College of Animal Experimentation (COBEA) and were approved by the Ethical Committee for Animal Research of the University of Uberaba, Brazil (protocol 0107/2009). The number of animals used was the minimum number necessary to show consistent effects of the drug treatments. At the end of the experiments, the animals were anesthetized with 60 mg/kg ketamine plus 7.5 mg/kg xylazine, and euthanized by anesthetic depth.

#### Writhing test induced by acetic acid

The antinociceptive effect was evaluated in mice using the writhing test induced by acetic acid according to the procedures previously described [27,28]. Animals were treated subcutaneously (s.c.) with the ethanolic extract of the bark of *M*. *tenuiflora* (50, 100 or 200 mg/kg) or the hexane, DCM, EtOAc or BuOH fractions of *M*. *tenuiflora* (100 mg/kg) 30 min prior to intraperitoneal (i.p.) injection of 0.6% acetic acid (0.1 mL/10 g, St. Louis, MO, USA). The negative control received only vehicle (negative control group, s.c.), and the positive control received indomethacin (positive control group, 10 mg/kg, i.p.). The mice were placed in separate boxes. After the administration of the acetic acid, the number of writhes and stretching movements (contraction of the abdominal musculature and extension of the hind limbs) was counted at 5 min intervals for a period of 30 min. Percentage of antinociception was calculated according to the following formula:

%AN={(control group average of the nociception–test group average of nociception)/(control group average of the nociception)}*100

#### Nociception induced by intraplantar injection of formalin

Formalin-induced hypernociception behavior was assessed as described previously [29]. Mice were allowed to acclimate for 15 min in a plastic box and pretreated 30 min prior to the injection with ethanol extract (50, 100 or 200 mg/kg, s.c.), vehicle (s.c.), indomethacin (10 mg/kg, i.p.), morphine (5 mg/kg, s.c.) or the hexane, DCM, EtOAc or BuOH fractions of *M*. *tenuiflora* (100 mg/kg, s.c.). Then, the mice were injected subcutaneously with 20 μl of 1.5% formaldehyde (St. Louis, MO, USA) in 0.9% saline into the plantar right hindpaws. The mice were observed for 30 min after the formalin injection, and the pain behavior was determined by the number of flinches, biting and licking of the injected paw counted during the observation period time. The acute phase (phase 1) was defined as 0–15 min after injection, and the persistent tonic phase (phase 2) was defined as 15–30 min after injection.

#### Paw edema induced by intraplantar injection of formalin

After the end of the experiment of nociception induced by intraplantar injection of formalin, both posterior paws were removed and weighed using an analytical balance. The contralateral paw was used as a control. The weight of the paw that did not receive the formalin injection was subtracted from the weight of the injected paw to determine edema formation. The results were express in grams (g). Percentage of antiedematogenic effect was also calculated according to the fowolling formula:

%AN={(control group average of the edema–test group average of edema)/(control group average of the edema)}*100

#### Evaluation of neutrophil migration

To investigate neutrophil migration to the peritoneal cavity, the ethanol extract (50, 100 or 200 mg/kg), vehicle, the hexane (200 mg/kg) or EtOAc (50 mg/kg) fractions of *M*. *tenuiflora* were administered subcutaneously 30 min prior to the administration of the inflammatory stimulus: an intraperitoneal injection of Cg at a dose of 500 μg/cavity [30]. The mice were sacrificed 4 h after Cg administration, and the peritoneal cavity cells were harvested by washing the cavity with 3 mL of phosphate-buffered saline (PBS) containing 1 mM EDTA. The recovered volume was similar in all experimental groups and equated to approximately 95% of the injected volume. Total counts were performed in a Newbauer chamber. Differential cell counts (100 cells in total) were performed on a cytocentrifuge (PRESVAC CT12, Curitiba, Brazil) using slides stained with Panoptic. The results are presented as the number of neutrophils per cavity.

#### Evaluation of vascular permeability

Vascular permeability was analyzed by the Evans Blue test [31]. Fifteen min prior to ethanol extract administration, Evans Blue (50 mg/kg) diluted in 50 μL of saline solution was intravenously injected into the ocular plexus. The ethanol extract, hexane, DCM, EtOAc or BuOH fractions of *M*. *tenuiflora* (100 mg/kg) or vehicle was administered subcutaneously 30 min prior to the inflammatory stimulus (intraperitoneal injection of Cg, 500 μg/cavity) [32]. The mice were sacrificed 4 h after Cg administration, and the peritoneal cavity was washed with 3 mL of PBS. The Evans blue content (protein extravasation) was calculated using an Evans blue standard curve, and the absorbance of each sample was measured at 620 nm using a spectrophotometer (Genesys, Thermo Scientific, Waltham, MA, USA).

#### Test of increasing pressure on paw

The hypernociceptive mechanical threshold was measured by the electronic Von Frey method as previously described [33]. Mice were placed in acrylic cages (12 x 20 x 17 cm) with wire grid floors in a quiet room 30 min before the experiment. The test consisted of evoking a hind paw flexion reflex with a hand-held force transducer adapted with a tip (0.8-mm^2^ tip diameter, electronic Von Frey, Insight Equipments©, Brazil). A tilted mirror placed under the grid provided a clear view of the mice hind paw. The investigator was trained to apply the tip in between the five distal footpads with a gradual increase in pressure. The stimulus was automatically discontinued and its intensity was recorded when the paw was withdrawn. The interval between two consecutive trials on the same paw was at least 1 min for a total of 8 trials per animal. The maximal force applied was 12 g. The endpoint was characterized by the removal of the paw in a clear flinch response.

The mice were pretreated with ethanol extract, the hexane, DCM, EtOAc or BuOH fractions of *M*. *tenuiflora* (100 mg/kg, s.c.) or vehicle (s.c.). After 30 min, the mice were injected with Cg (100 μg/paw, i.pl.) on the ventral surface of the hind paw. After three h, the Von Frey test was conducted. The results are expressed as the delta (Δ) withdrawal threshold (in g) calculated by subtracting the basal mean measurements from the mean measurements obtained 3 h after Cg i.p. injection.

#### Cytokine detection by ELISA

The mice were treated with the ethanol extract (100 mg/kg, s.c.) or vehicle (s.c.) after 30 min of Cg stimulus (500 μg/cavity, i.p.). The peritoneal exudate was collected to quantify cytokines after 4 h of treatment with the extract. TNF-α and IL-1β were determined by ELISA using the protocols supplied by the manufacturer (R & D Systems^®^, Minneapolis, MN, USA). The results are expressed in pg/mL. As a control, the concentrations of these cytokines were determined in animals that received saline injections.

#### Myeloperoxidase activity test

The extent of neutrophil accumulation in the paw was measured using the myeloperoxidase activity assay [30]. After the Von Frey test with the ethanolic extract of *M*. *tenuiflora* (100 mg/kg, s.c.) or vehicle (s.c.), the paw tissue was removed and homogenized in a pH 4.7 buffer (0.1 M NaCl, 0.02 M NaPO_4_, and 1.015 M NaEDTA), followed by centrifugation at 3000 x g for 15 min. The precipitate (pellet) was subjected to hypotonic lysis (1.5 mL of a 0.2% NaCl solution); after 30 seconds, an equal volume of a solution containing 1.6% NaCl and 5% dextrose was added. After further centrifugation, the pellet was resuspended in 0.05 M NaPO_4_ buffer (pH 5.4) containing 0.5% hexadecyltrimethylammonium bromide. Thereafter, the tissue was frozen by immersion in liquid nitrogen three times, centrifuged at 10,192 × g for 15 min and rehomogenized. Myeloperoxidase activity in the resuspended pellet was determined by the variation in the optical density at 450 nm using luminol (1.6 mM) and H_2_O_2_ (0.5 mM). The results were calculated by comparing the optical density of the paw tissue supernatant with a standard curve of the numbers of neutrophils (> 95% purity).

#### Mesenteric protein expression by Western blot analysis

The animals were previously pretreated with the ethanolic extract of *M*. *tenuiflora* (100 mg/kg, s.c.) or vehicle (s.c.), challenged after 30 min with Cg (i.p.) and sacrificed after 4 h. The mesentery was collected for the analysis of ICAM-1 protein expression. Protein quantification was conducted by the MicroBCA method. Briefly, the sample was homogenized in 400 μl of buffer (1% Triton X-100, 1 M NaF, 100 mM Nappi, 1 M Na_3_VO_4_, 1 mg/mL aprotinin, 1 mg/mL leupeptin, and 1 mg/mL phenylmethylsulfonyl fluoride) and centrifuged at 13,000 rpm at 4°C for 20 min. Equal amounts of protein were electrophoretically separated in a 10% SDS gel, followed by transfer to a nitrocellulose membrane. After transfer, the membrane was blocked with skim milk (5%) in TBS-T (20 mM Tris-HCl, pH 7.5, 500 mM NaCl, and 0.1% Tween 20) overnight at 4°C. Then, the membrane was incubated with a mouse anti-ICAM-1 antibody for 2 h at room temperature. The membrane was washed and incubated with a peroxidase-conjugated goat anti-mouse IgG antibody for 1 h at room temperature. Then, the membrane was washed and the ECL developer solution (Pierce) was applied. Images were captured using X-ray films (Kodak) to observe the presence of a band with a molecular weight of 85–110 kDa representing ICAM-1.

### Statistical analysis

The results were presented as the mean ± S.D. (n = 8 per experiment). Statistical comparisons between groups were conducted using analysis of variance (ANOVA) (one -way) followed by the Bonferroni test for multiple comparisons in the GraphPad PRISM^®^ version 5.00 program. The established level of significance was *P* < 0.05.

## Results

### Isolation of flavonoids

The flavonoids 5,4’-dihydroxy-7-methoxyflavanone (sakuranetin, **1**), 5,4’-dihydroxy-7-methoxyflavone (genkwanin, **3**), 5,6,4’-trihydroxy-7-methoxyflavone (sorbifolin, **4**), 5,4’-di-hydroxy-7,8-dimethoxyflavone (**5**), and 5,7,4’-trihydroxy-3-methoxyflavone (**6**) and the minor flavonols 5,7,4’-trihydroxy-6-methoxyflavonol (**7**), 5,6-dihydroxy-7,4’-dimethoxyflavonol (**8**) and 5-hydroxy-7,8,4’-trimethoxyflavonol (**9**) from the leaves of *M*. *tenuiflora* were identified by NMR, IR, UV and MS spectra data analysis and comparison with the literature (Fig 1). The ^1^H NMR and MS spectral data of compounds **1**–**9** are presented in the Supporting Information.

In previous studies dealing with this plant, isosakuranetin (5,4’-dihydroxy-7-methoxyflavanone, **2**) was identified as the main flavone present in the extract. In this study, this compound was not isolated. Moreover, the literature NMR data for these two isomers are confusing [16]. In addition to bidimensional NMR experiments, MS fragmentation also permitted the differentiation of isosakuranetin from sakuranetin. The fragments of the sililated derivative of isosakuranetin (especially the Retro Diels-alder at *m/z* 296) indicated that the flavanone A-ring did not bear a methoxyl group. In sakuranetin, this fragment was recorded at *m/z* 238 (Supporting Information).

### Effect on writhing induced by acetic acid

**Fig 2:**
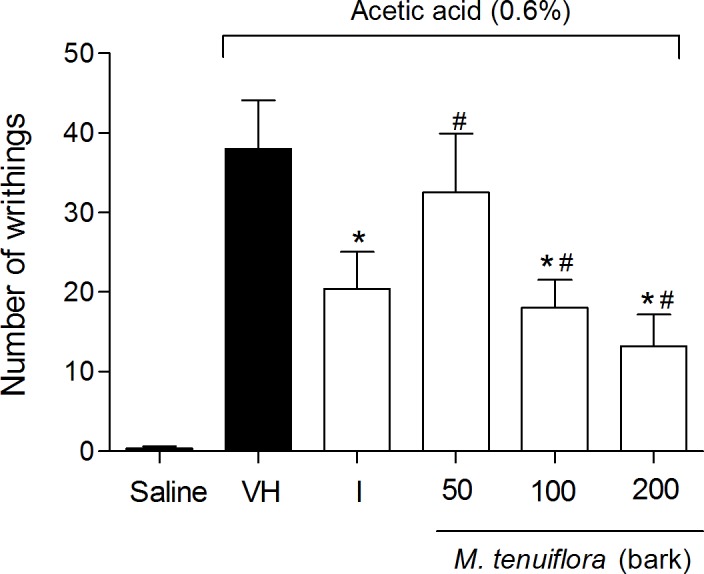
Effect of pretreatment of the mice with 50, 100 or 200 mg/kg of the ethanolic extract of *M*. *tenuiflora* bark on writhing induced by acetic acid (0.6%). VH is the vehicle group (negative control) and I is the positive control employing indomethacin (10 mg/kg, i.p.). The extract ethanol of *M*. *tenuiflora* bark was tested at doses of 50, 100 or 200 mg/kg (s.c.). The results are presented as the means ± S.D. of writhing in mice (n = 8). Statistical significance was calculated by ANOVA followed by Bonferroni's test. *P < 0.05 compared to the vehicle-negative control treated group. # *P* > 0.05 compared to the indomethacin—positive control group.

To evaluate the peripheral antinociceptive effect of the ethanolic extract of *M*. *tenuiflora* bark (EEMT), the acetic acid-induced writhing test was performed with 50, 100 and 200 mg/kg doses. The 100 and 200 mg/kg doses resulted in a significant reduction (*P* < 0.05) in the number of writhing episodes compared to the group treated with vehicle (negative control group) (Fig 2). In contrast, the 50 mg/kg dose was not able to significantly reduce the abdominal constriction response based on the mean number of writhes of the vehicle group (*P* < 0.05). The indomethacin, positive control, also reduced the writhes compared to vehicle group (*P* < 0.05). The EEMT at 50, 100 and 200 mg/kg showed similar antinociception compared to indomethacin (*P* > 0.05). Therefore, EEMT resulted in a dose–dependent inhibition of writhing in response to nociception.

### Effect on nociception induced by intraplantar injection of formalin

**Fig 3:**
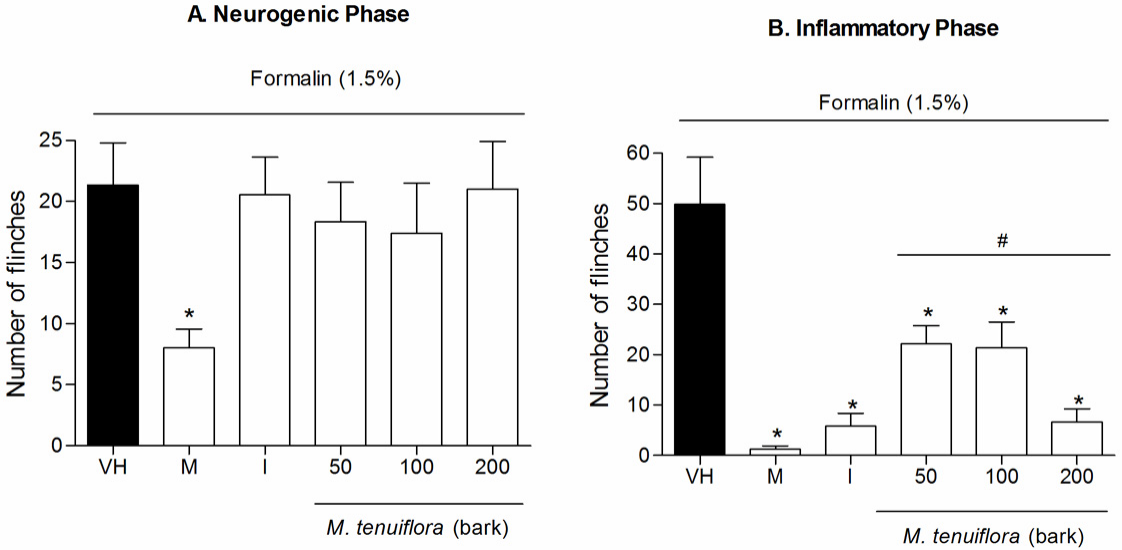
Effects of ethanolic extract of *M*. *tenuiflora* bark on the response to nociception induced by intraplantar formalin injection in mice during the neurogenic phase (3A) and inflammatory phase (3B). Morphine (M, 5 mg/kg, s.c.) and indomethacin (I, 10 mg/kg, i.p.) were the positive control group. VH is the vehicle group (negative control). The ethanolic extract of *M*. *tenuiflora* bark was tested at doses of 50, 100 or 200 mg/kg (s.c.). The results are presented as the mean ± S.D. (n = 8) of the number of flinches in the injected paw for a period of 30 min. Statistical significance was calculated by ANOVA followed by Bonferroni's test. *P < 0.05 compared to the vehicle-negative control treated group. # *P* > 0.05 compared to the indomethacin or morphine—positive control groups.

We used the formalin model to confirm the antinociceptive effect of EEMT. With this model, we can access the antinociceptive effect and also evaluate a possible anti-inflammatory effect (Fig 3). The administration of the ethanol extract of the bark at doses of 50, 100 and 200 mg/kg 30 min before formalin nociception failed to inhibit the formalin-induced nociception produced during the neurogenic phase in the animal paw (*P* > 0.05) (Fig 3A). Indomethacin also did not showed reduction of nociception (*P* > 0.05), and only morphine showed good activity in reduction of flinches compared to negative control (*P <* 0.05). However, flinches in the inflammatory phase (Fig 3B) were significantly decreased by all three doses of the extract compared to the vehicle treated group (*P* < 0.05) in a dose–dependent manner. Therefore, all three doses of EEMT showed inhibition of nociception similar to the morphine and the indomethacin groups (*P* > 0.05) (Fig 3B).

### Effect on edema induced by intraplantar injection of formalin

**Fig 4:**
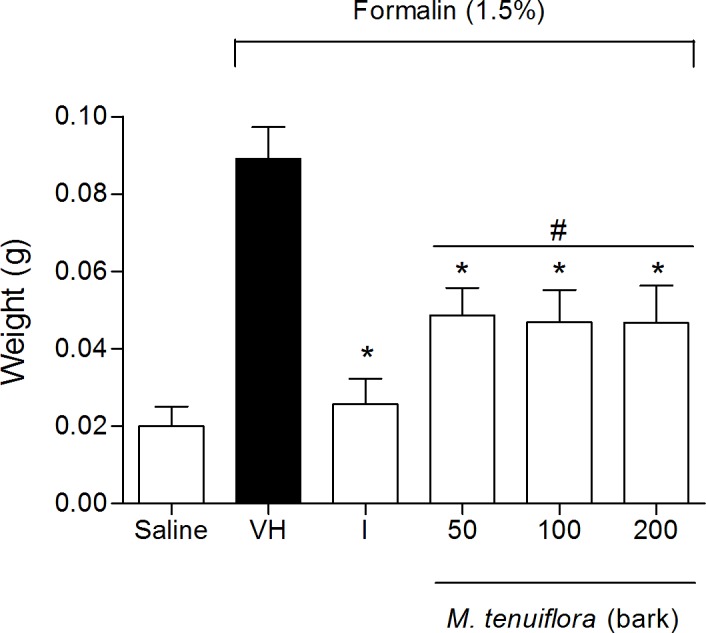
Effect of ethanolic extract of *M*. *tenuiflora* bark on the induction of paw edema in mice pretreated with an intraplantar injection of 1.5% formalin. VH is the vehicle group (negative control). The positive control was indomethacin (10 mg/kg, i.p.). The ethanol extract of *M*. *tenuiflora* bark was tested at doses of 50, 100 or 200 mg/kg (s.c.). The results are presented as the mean ± S.D. (n = 8). Statistical significance was calculated by ANOVA followed by Bonferroni's test. *P < 0.05 compared to the vehicle-negative control treated group. # *P* > 0.05 compared to the indomethacin—positive control group.

The treatment with the extract also significantly reduced the edema formation induced by formalin injection in the paws of mice compared to the vehicle group (*P* < 0.05), indicating that the antiedematogenic activities of EEMT were similar to the group treated with the standard drug indomethacin (*P* > 0.05) (Fig 4).

### Effect on the migration of neutrophils to the peritoneal cavity

**Fig 5:**
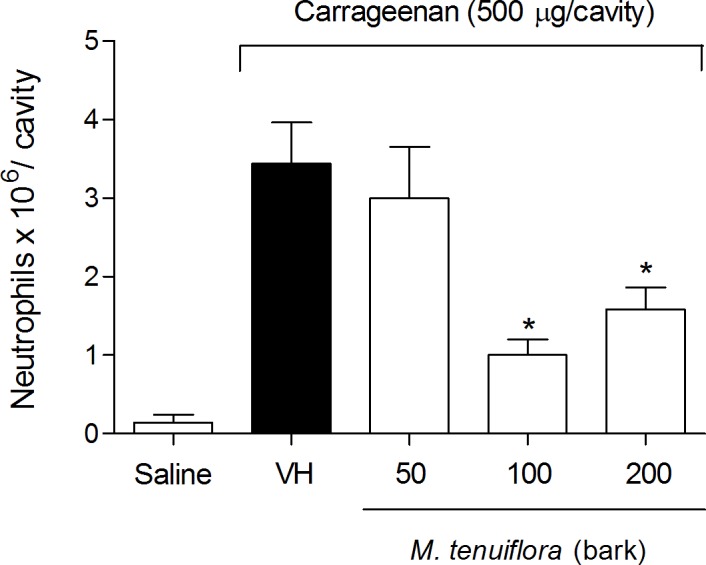
Effect of ethanolic extract of *M*. *tenuiflora* bark on neutrophil migration into the peritoneal cavity of mice pretreated subcutaneously 30 min prior to Cg injection (500 μg/cavity) to induce peritonitis. VH is the vehicle group (negative control). The ethanol extract of *M*. *tenuiflora* bark was tested at doses of 50, 100 or 200 mg/kg (s.c.). The results are presented as the mean ± S.D. (n = 8). Statistical significance was calculated by ANOVA followed by Bonferroni's test. *P < 0.05 compared to the vehicle-negative control treated group.

The possible anti-inflammatory effect observed in the second phase of formalin-induced licking led us to evaluate the effects of EEMT in other models of inflammation *in vivo*. First, the inhibition of Cg-induced neutrophil migration to the peritoneal cavity was tested after 4 h (Fig 5). Pre-treatment with 100 and 200 mg/kg (s.c.) of the extract promoted a decrease in neutrophil migration (*P* < 0.05) to the peritoneal cavity. In contrast, the 50 mg/kg dose was not able to reduce the migration of neutrophils to the cavity (*P* > 0.05). Because the 100 mg/kg dose showed better activity in the anti-inflammatory test of inhibition of neutrophil migration, we tested this dose in the other anti-inflammatory models.

### Evaluation of the effect on vascular permeability

**Fig 6:**
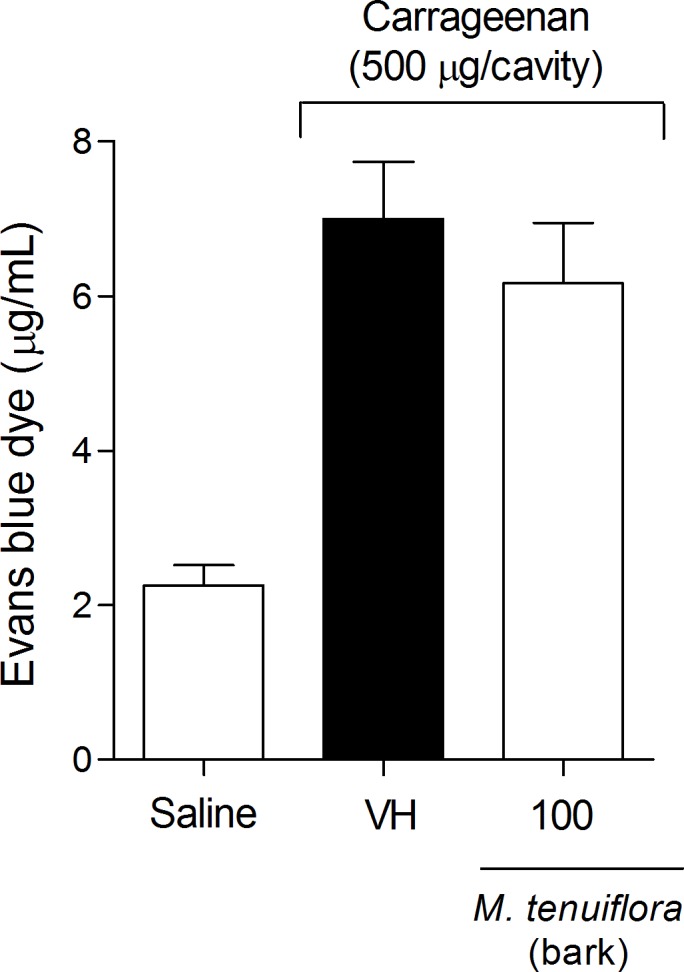
Effect of ethanolic extract of *M*. *tenuiflora* bark on vascular permeability using the Evans blue test. VH is the vehicle group (negative control). The ethanol extract of *M*. *tenuiflora* bark was tested at the 100 mg/kg (s.c.) dose. The results are presented as the mean ± S.D. (n = 8). Statistical significance was calculated by ANOVA followed by Bonferroni's test. *P < 0.05 compared to the vehicle-negative control treated group.

Another approach to evaluate the anti-inflammatory response was to analyze vascular extravasation identified using Evans blue dye (Fig 6). The *M*. *tenuiflora* extract showed a slight reduction in vascular permeability at the dose tested (100 mg/kg), but was not significantly different from the vehicle group (*P* > 0.05). Therefore, the 100 mg/kg dose may not have been sufficient to induce an obvious result.

### Effect on the inhibition of Cg-induced hyperalgesia

**Fig 7:**
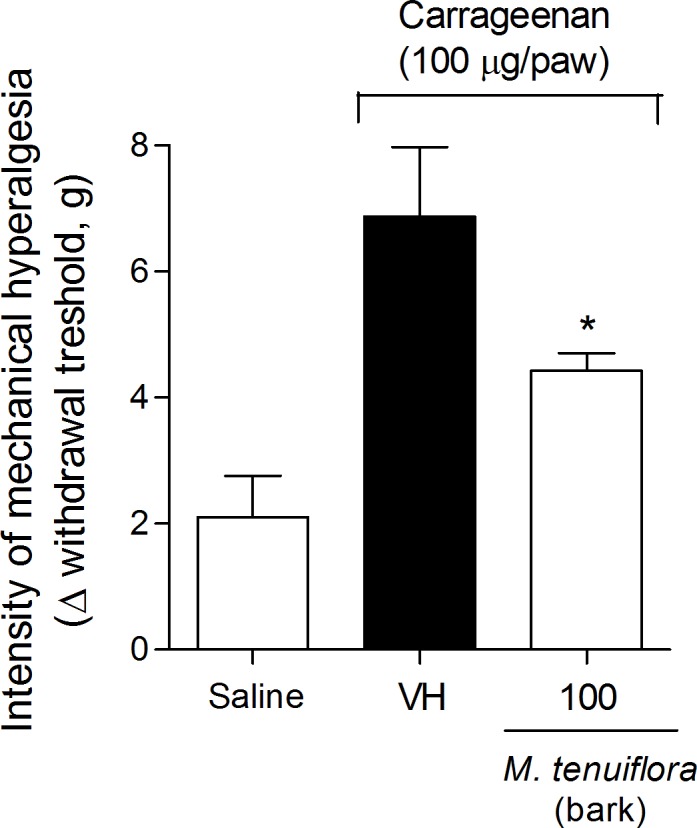
Effect of ethanolic extract of *M*. *tenuiflora* bark on the inhibition of Cg-induced hyperalgesia. VH is the vehicle group (negative control). The ethanol extract of *M*. *tenuiflora* bark was tested at a dose of 100 mg/kg (s.c.). The results are presented as the mean ± S.D. (n = 8). Statistical significance was calculated by ANOVA followed by Bonferroni's test. *P < 0.05 compared to the vehicle-negative control treated group.

The inflammatory nociception induced by Cg was performed using the Von Frey test to evaluate tissue sensitivity to mechanical stimulation (mechanical hyperalgesia). Cg injection significantly decreased the basal values measured by electronic Von Frey mechanical stimulation. Treatment with 100 mg/kg EEMT significantly inhibited the Cg-induced mechanical hyperalgesia at 3 h (*P* < 0.05) (Fig 7).

### Effect on TNF-α and IL-10 production in the peritoneal exudate

**Fig 8:**
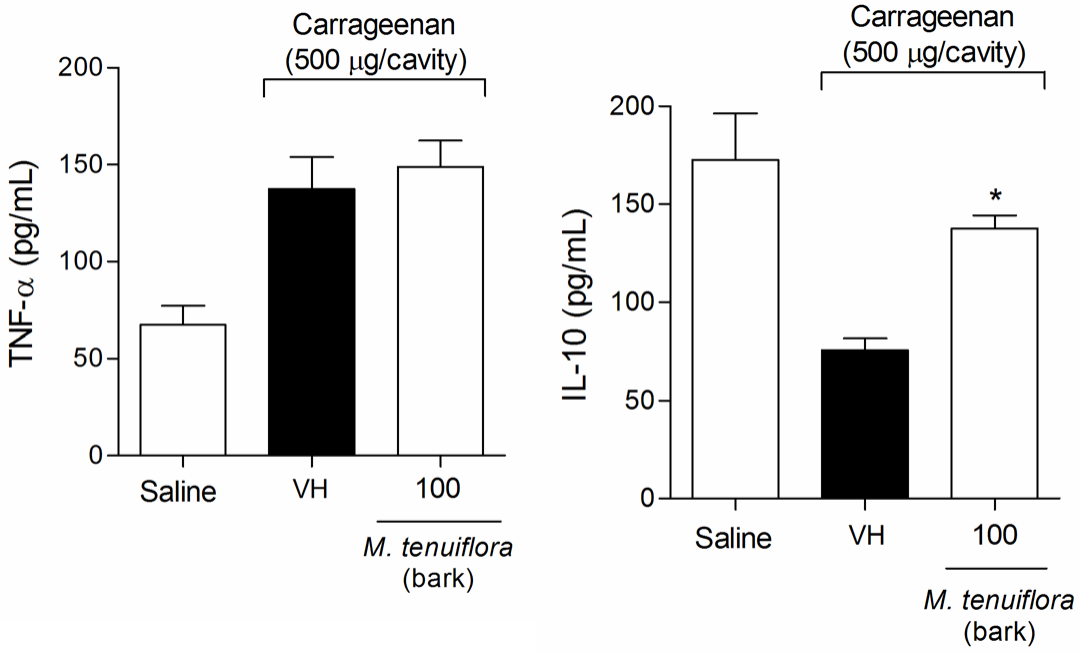
Effect of the ethanolic extract of *M*. *tenuiflora* bark on Cg-induced TNF-α (A) and IL-10 (B) production in the peritoneal exudate. VH is the vehicle group (negative control). The ethanol extract of *M*. *tenuiflora* bark was tested at a dose of 100 mg/kg (s.c.). The results are presented as the mean ± S.D. (n = 8). Statistical significance was calculated by ANOVA followed by Bonferroni's test. *P < 0.05 compared to the vehicle-negative control treated group.

Mice were treated with EEMT (100 mg/kg) 30 min before Cg i.p. injection. Samples from the peritoneal exudate were collected 4 h after Cg injection to measure TNF-α (Fig 8A) and IL-10 (Fig 8B). Cg significantly increased the pro-inflammatory cytokine TNF-α and reduced the IL-10 levels in the peritoneal exudate. Treatment with 100 mg/kg EEMT did not affect the TNF-α levels in the peritoneal exudate. However, 100 mg/kg EEMT increased the levels of the anti-inflammatory IL-10 in the peritoneal cavity 4 h after Cg administration.

### Effect on myeloperoxidase activity (MPO)

**Fig 9:**
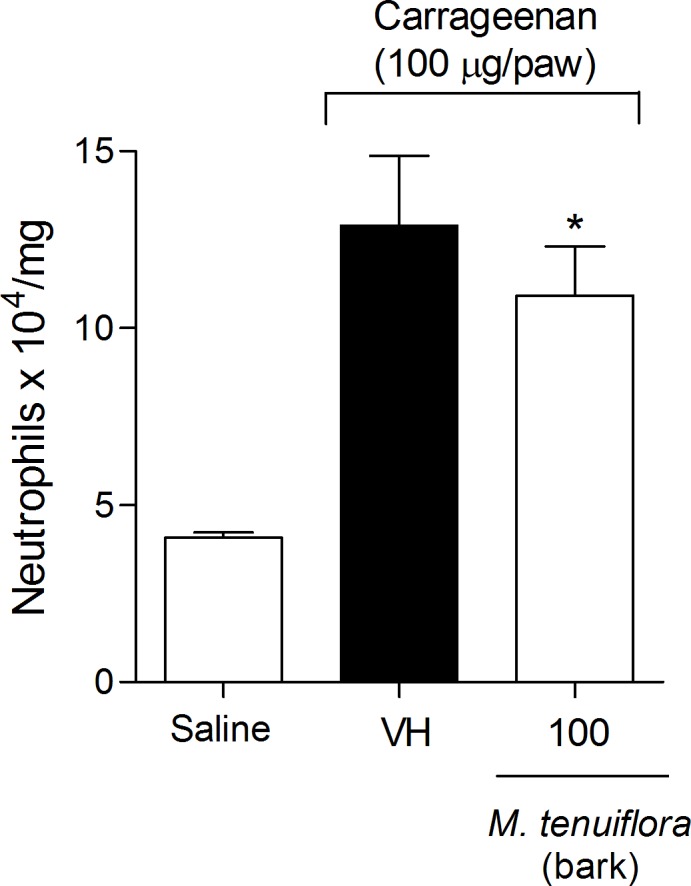
Effect of the ethanolic extract of *M*. *tenuiflora* bark on the inflammatory nociception association with the Cg-induced migration of neutrophils into the plantar tissue evaluated by determining myeloperoxidase activity (MPO). VH is the vehicle group (negative control). The ethanolic extract of *M*. *tenuiflora* bark was tested at the dose of 100 mg/kg (s.c.). The results are presented as the mean ± S.D. (n = 8). Statistical significance was calculated by ANOVA followed by Bonferroni's test. *P < 0.05 compared to the vehicle-negative control treated group.

MPO (myeloperoxidase) is an enzyme found primarily in neutrophils, and an increase in enzyme activity is an indirect indication of the presence of a greater number of neutrophils in the inflamed tissue. A small but significant inhibition of neutrophil migration into the plantar tissue was observed following Cg induction in animals treated with EEMT 100 mg/kg (*P* < 0.05) based on measurement of the MPO activity (Fig 9).

### ICAM-1 expression

**Fig 10:**
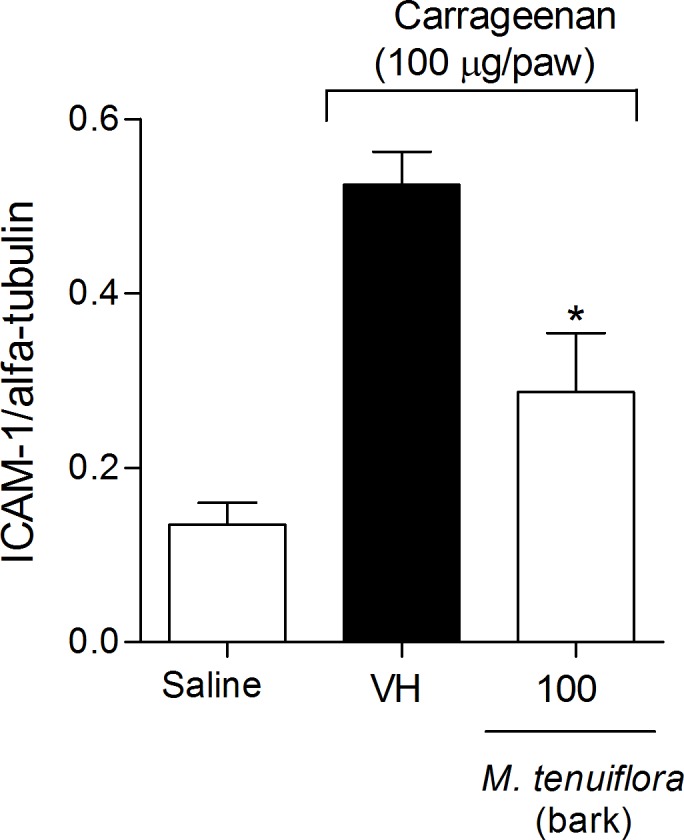
Effect of the ethanolic extract of *M*. *tenuiflora* bark on ICAM-1 expression in the mesentery of mice. VH is the vehicle group (negative control). The ethanolic extract of *M*. *tenuiflora* bark was tested at the dose of 100 mg/kg (s.c.). The results are presented as the mean ± S.D. (n = 8). The optical density of the bands for ICAM-1 was normalized to α-tubulin expression. Statistical significance was calculated by ANOVA followed by Bonferroni's test. *P < 0.05 compared to the vehicle-negative control treated group.

Another test conducted to elucidate the mechanism of action of EEMT on inflammation was the evaluation of the expression of intercellular adhesion molecule-1 endothelial (ICAM-1). We used Western blotting to assess ICAM-1 expression in the mesentery of the mice based on a standard curve of α-tubulin expression. A dose of 100 mg/kg EEMT decreased ICAM-1 expression in the mesentery compared to the vehicle group (*P* < 0.05) (Fig 10).

### Effect of the fractions on acetic acid-induced writhing

To attempt to identify the compound(s) responsible for the antinociceptive and anti-inflammatory activities demonstrated by the *M*. *tenuiflora* (bark) ethanolic extract, tests were performed with the fractions obtained after solvent partition between hexane, DCM, EtOAc and BuOH.

**Fig 11:**
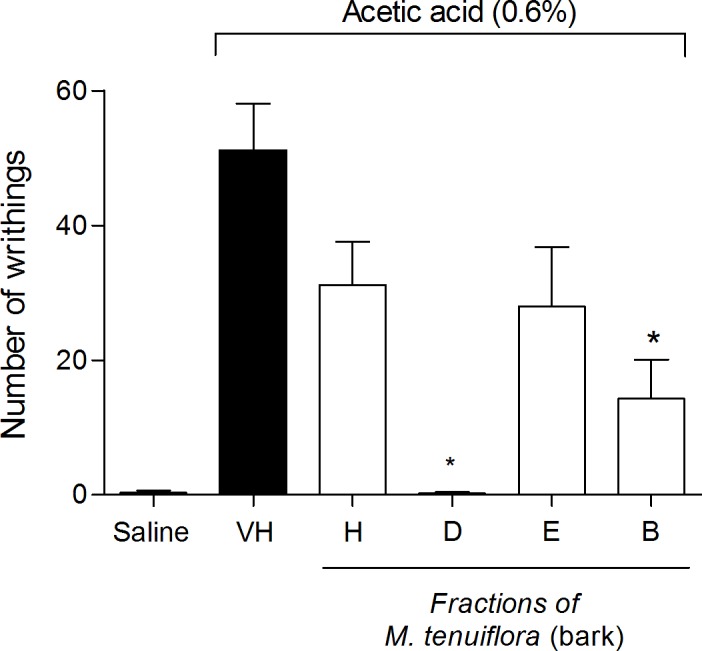
Effect of pretreatment of the mice with the hexane (H), DCM (D), ETOAc (E) and BuOH (B) (100 mg/kg) soluble fractions of the *M*. *tenuiflora* extract on acetic acid (0.6%)-induced writhing. VH is the vehicle group (negative control). The results are presented as the mean ± S.D. (n = 8). Statistical significance was calculated by ANOVA followed by Bonferroni's test. *P < 0.05 compared to the vehicle-negative control treated group.

The four soluble fractions were tested at doses of 100 mg/kg in the writhing test (Fig 11). Only the DCM and BuOH fractions significantly reduced the writhing in mice induced by 0.6% acetic acid compared with the vehicle group (*P* < 0.05).

### Effect of the fractions on nociception induced by intraplantar formalin

**Fig 12:**
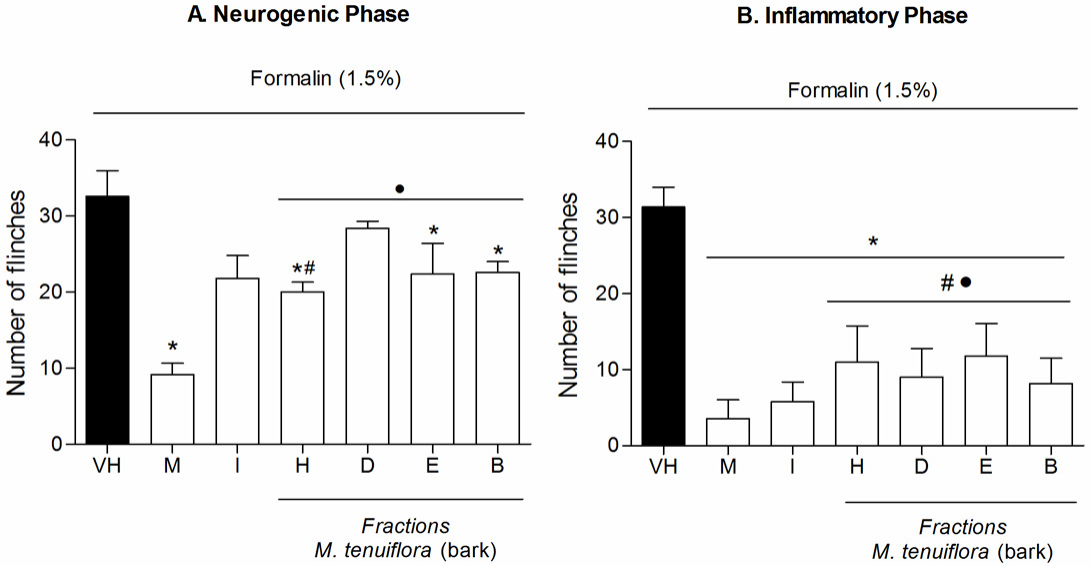
Effect of the hexane (H), DCM (D), EtOAc (E) and BuOH (B) *M*. *tenuiflora* bark extract fractions on the nociception induced by intraplantar formalin injection in mice. Neurogenic phase (Fig 12A) and inflammatory phase (Fig 12B). Morphine (M, 5 mg/kg, s.c.) and indomethacin (I, 10 mg/kg, i.p.) were the positive control groups. The fractions were tested at 100 mg/kg. VH is the vehicle group (negative control). The results are presented as the mean ± S.D. (n = 8). Statistical significance was calculated by ANOVA followed by Bonferroni's test. *P < 0.05 compared to the vehicle-negative control treated group. # *P* > 0.05 compared to the morphine-treated group. • *P* > 0.05 compared to the indomethacin-treated group.

The four fractions at doses of 100 mg/kg (Fig 12) were also evaluated in the formalin test. During the first phase (neurogenic phase–Fig 12A), the hexane, EtOAc and BuOH fractions resulted in a reduction in the number of flinches compared to the vehicle group (*P* < 0.05). Morphine showed great antinociception (*P* < 0.05), while indomethacin was not able to reduce nociception at first phase. Only hexane treatment reduced nociception similar to morphine (*P* > 0.05) and hexane, dicloromethane, EtOAc and BuOH fractions showed reduction similar to indomethacin (*P* > 0.05). During the second phase (inflammatory phase–Fig 12B), all four fractions reduced the flinches provoked by formalin and showed similar antinociception compared to the standard drugs morphine and indomethacin (*P* > 0.05).

### Effect of the fractions on paw edema after administration of formalin

**Fig 13:**
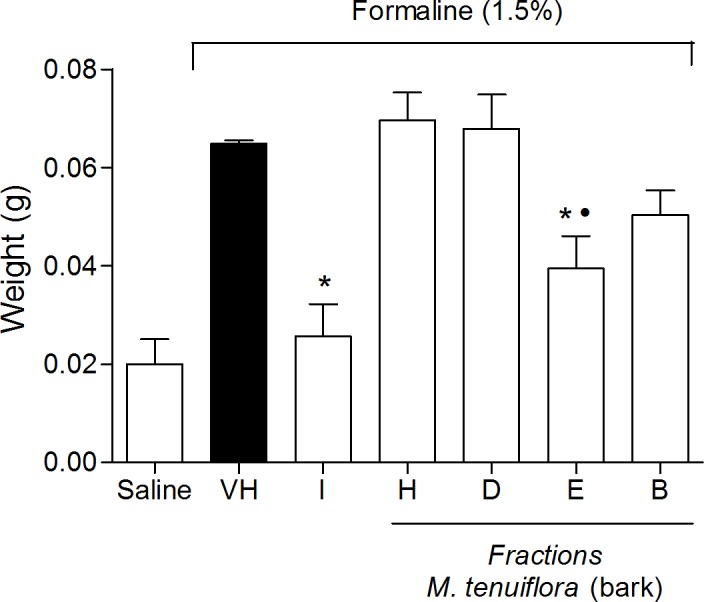
Effect of fractions of H-hexane, D-DCM, E-EtOAc and B-BuOH at 100 mg/kg of *M*. *tenuiflora* bark extract on paw edema after administration of formalin 1.5% (i.pl). VH is the vehicle group (negative control). Indomethacin (I, 10 mg/kg, i.p.) was the positive control group. Results are presented as mean ± S.D. (n = 8). Statistical significance was calculated by ANOVA followed by Bonferroni's test. *P < 0.05 compared to the vehicle-negative control treated group. •*P* > 0.05 compared to indomethacin-treated group.

Next, we evaluated paw edema after formalin injection (Fig 13). Only the EtOAc fraction resulted in a reduction in paw edema after treatment (*P* < 0.05). Indomethacin showed a good reduction in nociception in mice, a percentage of antiedematogenic of 60.46 ± 0.01%, compared to the negative control (*P* < 0.05). The EtOAc fraction demonstrated a percentage of antiedematogenic of 39.20 ± 0.01% compared to the negative control, not differing statistically to the antiedematogenic effect showed by indomethacin (*P* > 0.05).

#### Effect of the fractions on neutrophil migration to the peritoneal cavity

**Fig 14:**
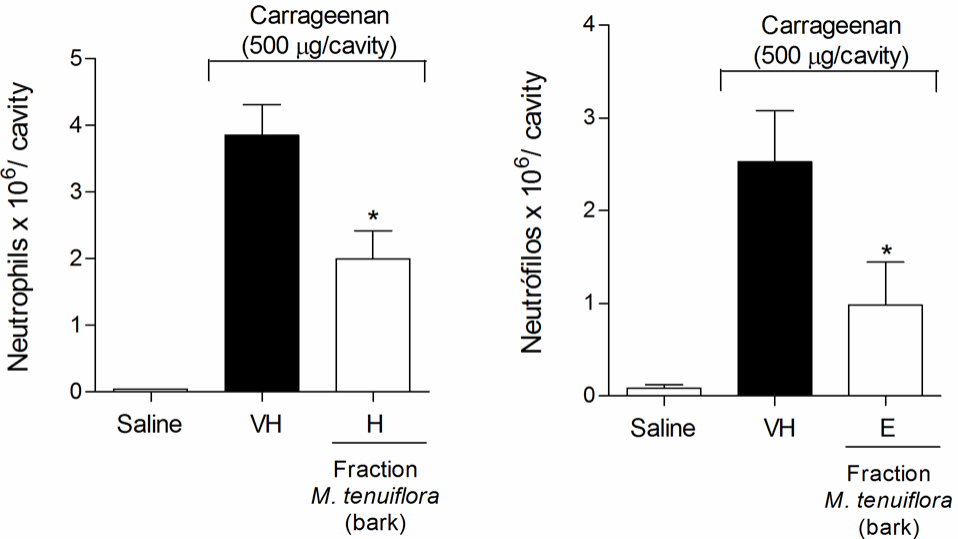
Effect of the hexane (H) (200 mg/kg) and EtOAc (E) (50 mg/kg) *M*. *tenuiflora* bark extract fractions on neutrophil migration to the peritoneal cavity of mice after Cg administration. VH is the vehicle group (negative control). The results are presented as the mean ± S.D. (n = 8). Statistical significance was calculated by ANOVA followed by Bonferroni's test. *P < 0.05 compared to the vehicle-negative control treated group.

The four fractions at doses of 50, 100 and 200 mg/kg were used in tests to verify the anti-inflammatory activities. Only the hexane fraction at a dose of 200 mg/kg (Fig 14A) and the EtOAc fraction at a dose of 50 mg/kg (Fig 14B) were able to reduce neutrophil migration to the peritoneal cavity after Cg administration (*P* < 0.05). The other fractions (DCM and BuOH) did not show a reduction in neutrophil migration activity (*P* > 0.05). None of the fractions showed inhibitory activity on neutrophil migration compared to the vehicle treated group at the 100 mg/kg dose (*P* > 0.05).

### Effect of the fractions on the Evans blue and Von Frey tests

**Fig 15:**
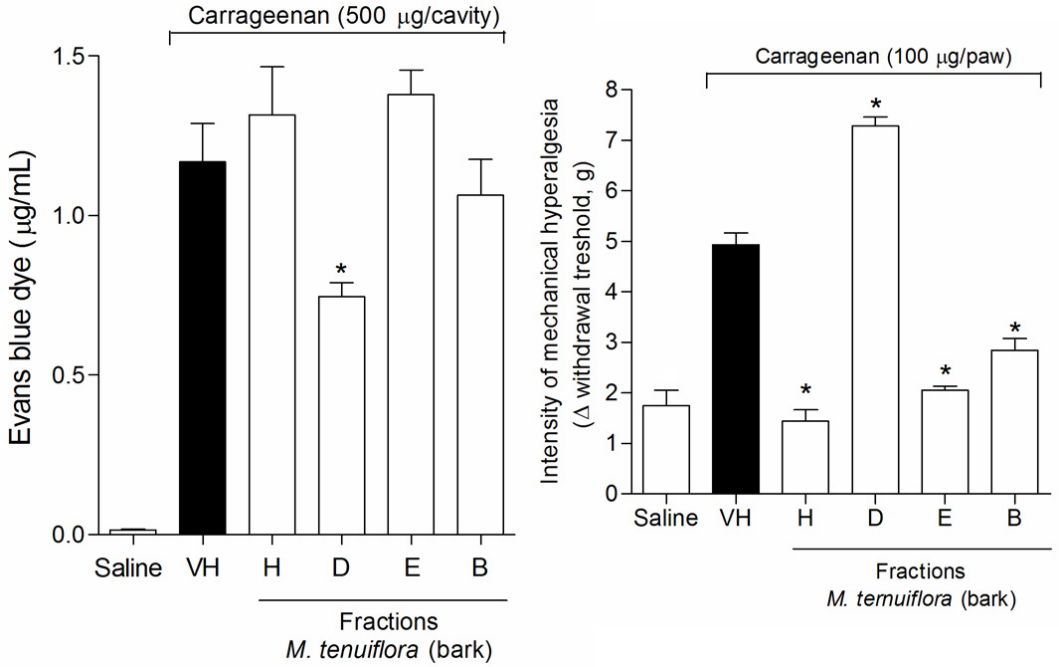
Effect of the hexane (H), DCM (D), EtOAc (E) and BuOH (B) (100 mg/kg) soluble fractions of *M*. *tenuiflora* bark in the Evans blue (A) and Von Frey tests (B). VH is the vehicle group (negative control). The results are presented as the mean ± S.D. (n = 8). Statistical significance was calculated by ANOVA followed by Bonferroni's test. *P < 0.05 compared to the vehicle-negative control treated group.

Cg injection increased vascular permeability, promoted an increase in the peritoneal exudate in the Evans blue test (Fig 15A), and significantly decreased the basal values measured by electronic Von Frey mechanical stimulation (Fig 15B). In the Evans blue test, only the DCM fraction (100 mg/kg) reduced the peritoneal exudate formed after Cg injection. In contrast, the other fractions showed a reduction in hypernociception at a 100 mg/kg dose in the Von Frey test; only the DCM fraction showed pro-inflammatory activity at this dose, resulting in a significant reduction in the basal values (*P* < 0.05).

### Effect of sakuranetin and synthetized isosakuranetin on the writhing test and on the recruitment of neutrophils to the peritoneal cavity

**Fig 16:**
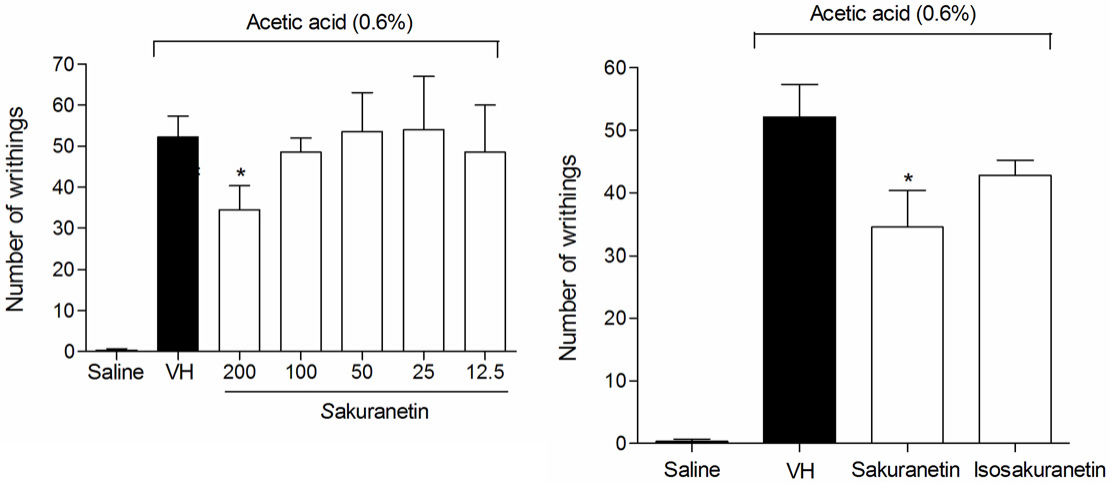
Effect of sakuranetin and synthetized isosakuranetin at a 200 mg/kg dose on acetic acid (0.6%)-induced writhing. Sakuranetin was tested at different doses (12.5–200 mg/kg) and compared to isosakuranetin. VH is the vehicle group (negative control). The results are presented as the mean ± S.D. (n = 8). Statistical significance was calculated by ANOVA followed by Bonferroni's test. *P < 0.05 compared to the vehicle-negative control treated group.

In an attempt to determine which compound or group of compounds was responsible for the antinociceptive and anti-inflammatory activity, two tests were performed with sakuranetin isolated from the DCM soluble fraction from the ethanolic extract of *M*. *tenuiflora* leaves. First, sakuranetin was used in the writhing test at different doses (12.5, 25, 50, 100 and 200 mg/kg) (Fig 16A). The 200 mg/k dose resulted in a significant reduction in the number of writhes compared to the group treated with the vehicle (*P* < 0.05). The test was also conducted with the synthesized isosakuranetin, which did not show a reduction in nociception (writhes). When both sakuranetin and isosakuranetin were compared at doses of 200 mg/kg, sakuranetin showed a better result than the synthesized isosakuranetin (*P* < 0.05) (Fig 16B).

**Fig 17:**
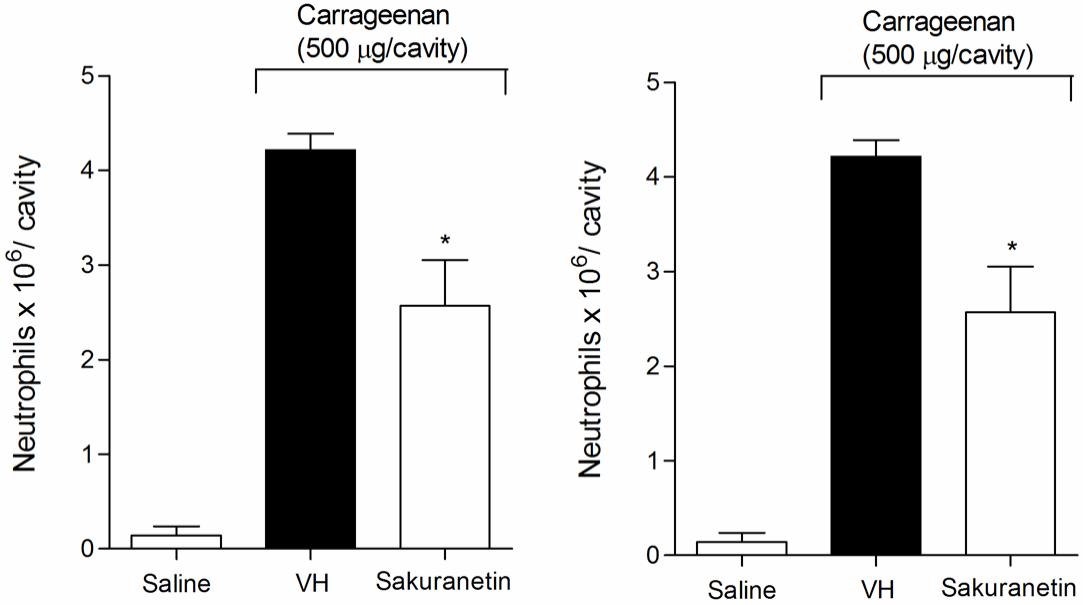
Effect of sakuranetin and synthetized isosakuranetin at a dose of 200 mg/kg on neutrophil recruitment to the peritoneal cavity of mice pretreated prior with Cg (500 μg/ cavity). VH is the vehicle group (negative control). The results are presented as the mean ± S.D. (n = 8). Statistical significance was calculated by ANOVA followed by Bonferroni's test. *P < 0.05 compared to the vehicle-negative control treated group.

In the test of neutrophil recruitment to the peritoneal cavity after Cg administration, the group treated with sakuranetin (200 mg/kg) showed a lower quantity of neutrophils compared to the vehicle-treated group (*P* < 0.05). Moreover, synthetized isosakuranetin (200 mg/kg) did not significantly reduce neutrophil levels in the peritoneal cavity of mice (*P* > 0.05) (Fig 17).

## Discussion

Pain is one of the most pervasive problems in our society and has high social and economic impacts [34]. During inflammation, several mediators can activate and/or sensitize nociceptive fibers and mediators; these mediators are also involved in edema formation and leukocyte infiltration [35]. Several analgesics are used to treat a wide range of painful and inflammatory conditions, including non-steroidal anti-inflammatory drugs (NSAIDs) [36], glucocorticoids [37] and opioids [38]. Despite the high diversity of available anti-inflammatory and analgesic drugs, their side effects and the ineffectiveness of some drugs for some conditions require the continuous search for new drugs [39]. Investigating plant–derived products is a vital way to discover effective and less toxic drugs [40]. The interest in *M*. *tenuiflora* has increased considerably throughout the world because different experimental studies have confirmed its reported therapeutic properties.

In this study, we demonstrated the peripheral antinociceptive and anti-inflammatory effects of the ethanolic extract and fractions of *M*. *tenuiflora* bark and investigated the activities of sakuranetin, a compound isolated from EEMT, and isosakuranetin, a product from *M*. *tenuiflora* synthesis.

In the *in vivo* antinociceptive experiments, EEMT demonstrated good dose–dependent antinociceptive activity in acetic acid–induced abdominal constriction and the second phase of the formalin test. The acetic acid–induced writhing method is widely used for the evaluation of peripheral antinociceptive activity and as a screening test for the assessment of new analgesic or anti-inflammatory agents [41]. Because acetic acid acts by inducing the release of mediators that can stimulate nociceptive neurons sensitive to non-steroidal anti-inflammatory drugs and narcotics [42], the constriction induced by acetic acid is considered to be a non–selective antinociceptive model. The EEMT treatment resulted in writhing inhibition rates of approximately 52.63 ± 9.09 and 65.20 ± 10.25% (percentage of antinociception) at doses of 100 and 200 mg/kg, respectively. The results of this writhing test alone do not indicate whether the antinociceptive effect was central or peripheral.

We used the formalin test to evaluate the neurogenic and inflammatory antinociceptive action of the ethanolic extract. The noxious stimulus induced by formalin in the paws of mice induced a biphasic response. The first phase occurred from 0 to 5 min of formalin-induced behavior and reflected the direct activation of the A-delta and C afferent fibers (also called the neurogenic phase). The second phase lasted from 20 to 30 min and was called the inflammatory phase; this phase reflects both ongoing peripheral sensory input and central sensitization. Centrally acting drugs such as narcotics inhibit the two phases of the nociceptive response equally [43], whereas drugs with peripheral action, such as aspirin and dexamethasone, inhibit only the second phase [44,45]. The potential antinociceptive effect from EEMT was confirmed by the reduction in the flinches only in the second phase by the three doses tested (50, 100 and 200 mg/kg). The higher dose (200 mg/kg) showed remarkable antinociceptive activity that was similar to the standard drug morphine. The edema promoted by formalin was reduced by approximately 50% by the three doses tested (50, 100 and 200 mg/kg), which was similar to the standard drug indomethacin. These peripheral antinociceptive activities indicate the potential anti-inflammatory effect of EEMT.

We analyzed the inhibition of neutrophil migration to the peritoneal cavity and performed the Evans Blue and Von Frey tests to follow up the investigation of the anti-inflammatory properties of the ethanol extract of *M*. *tenuiflora*. These effects were provoked by the administration of Cg, which induces the formation of bradykinin and a subsequent release of the proinflammatory cytokine cascade, thereby triggering the release of PGs and sympathetic amines [46,47].

Polymorphonuclear cells are the main cellular components that migrate to the acute inflammatory sites induced by Cg [42]. Cg-induced neutrophil migration to the peritoneal cavity is an acute inflammation model that allows the quantification of leukocytes that migrate to the peritoneal cavity under the action of chemotactic agents (mainly leukotrienes and interleukins) released by resident cells susceptible to nonsteroidal anti-inflammatory action [48]. The number of total leukocytes in the peritoneal cavity was evidently suppressed by the dose of 100 mg/kg to approximately 73% of the vehicle level.

A dose of 100 mg/kg EEMT was not able to reduce the vascular extravasation identified with the Evans blue dye. Evans blue dye forms a stable complex with plasma proteins, which overflow when there is an increase in vascular permeability during the inflammation process. The 100 mg/kg dose was most likely not sufficient to induce a significant decrease in extravasation.

The last test of nociception with EEMT was the Von Frey electronic test, which was performed to evaluate tissue sensitivity to mechanical stimulation. The 100 mg/kg EEMT dose significantly inhibited Cg-induced mechanical hyperalgesia, which results mainly from the sensitization of primary afferent neurons and is detected as a decrease of the nociceptive threshold in animal models [46]. They hyperalgesia is induced by inflammatory mediators, such as prostaglandins and sympathetic amines, that directly sensitize the peripheral nociceptive neurons [49]. The release of these direct-acting hyperalgesic mediators is generally preceded by a cascade of cytokines [50] that activate the second messenger pathways (cAMP, protein kinase A, and PKC) responsible for lowering the nociceptor threshold and increasing neuronal membrane excitability. In this state, nociceptor activation and impulse transmission by the primary nociceptive neurons are facilitated [51], and EEMT can interfere with these pathways.

Another important event that mediates inflammatory hypernociception is neutrophil migration. Neutrophil migration plays an important role in Cg-induced hyperalgesia because these cells provide an important source of the superoxide anion and other hyperalgesic mediators, such as PGE2 [52]. Investigations whether EEMT could reduce the number of neutrophils in the paws of mice after the administration of Cg were performed. Myeloperoxidase is a marker for the tissue content of polymorphonuclear neutrophils because myeloperoxidase activity is correlated with leucocyte infiltration in inflamed regions [53]. EEMT at a dose of 100 mg/kg was able to reduce the myeloperoxidase levels, which reflect the number of activated neutrophils at the local inflammation site.

If EEMT inhibited the migration of leukocytes to the peritoneal cavity, EEMT may directly influence the production of cytokines involved in inflammation. TNF-α and IL-1β are crucial mediators after tissue injury that directly chemoattract neutrophils and activate resident inflammatory cells, further amplifying the production of chemotactic molecules [54,55]. EEMT did not alter the TNF-α levels in the peritoneal exudate 4 h after Cg administration. However, 100 mg/kg EEMT increased the level of IL-10, which is one of the most potent and significant anti-inflammatory cytokines. The other profound effect of IL-10 is to inhibit the production of proinflammatory cytokines and mediators by macrophages [56]. The major inflammatory cytokines (IL-1, IL-6, IL-12, and TNF-α) are all dramatically repressed following exposure to IL-10 through the suppression of NF-κB activation. IL-10 can further inhibit inflammation by increasing the release of the IL-1 receptor antagonist by macrophages [56]. Some of the mechanisms of EEMT involved in the inhibition of neutrophil migration to the peritoneal cavity or the paws could be a result of the higher levels of IL-10. Increased IL-10 may influence the reduction of the production of the inflammatory cytokines IL-1 or IL-6 because the extract at the dose (100 mg/kg) tested did not interfere with TNF-α production.

Another possibility is that EEMT reduces the migration of neutrophils to the peritoneal cavity by interfering with adhesion molecules such as ICAM, which plays an important role in leukocyte migration to inflammatory sites. ICAM-1 is expressed on endothelial cells and is one of the major cell surface glycoproteins that contribute to cell adhesion processes [57]; it can be significantly induced in response to proinflammatory mediators such as TNF-α and IL-1β [58]. The association of ICAM-1 and the CD18 integrin allows the adhesion of neutrophils to the blood vessel wall, which is a prerequisite for efficient transendothelial migration [59]. Thus, the anti-inflammatory activity *M*. *tenuiflora* may be related to the reduction of neutrophil migration to the peritoneal exudate and to intraplantar tissue provoked by Cg.

EEMT showed important anti-inflammatory potential, mainly through mechanisms including interference with leucocyte transendothelial migration and accumulation at the local site of inflammation. Moreover, the effective antinociceptive dose of BHE (100 mg/kg) was relatively low compared with other plant extracts with analgesic activity [40, 60]. At the doses tested, EEMT appeared to act similarly to non-steroidal anti-inflammatory drugs (NSAIDs) by reducing nociception during the second phase of formalin-induced nociceptive behavior in mice and the migration of neutrophils to the peritoneal cavity.

The four fractions obtained through the partition of the ethanolic extract of the powder of *M*. *tenuiflora* bark, leaves and branches produced with the organic solvents hexane, DCM, EtOAc and BuOH produced varying yields. The extracts with the highest polarity (those obtained with EtOAc and BuOH from the barks and branches) showed the highest yields. From the leaves, the highest yields were obtained from the extracts with the lowest polarity (hexane and DCM). In an attempt to identify the compound(s) responsible for the antinociceptive and anti-inflammatory activities demonstrated by EEMT, the same tests performed with EEMT were performed with the fractions obtained after solvent partition with hexane, DCM, EtOAc and BuOH.

As shown in the present study, the hexane (H), DCM (D), ethyl-acetate (E) and BuOH (B) fractions obtained from the EEMT also showed anti-inflammatory and antinociceptive effects in the different tests. Thus, it is possible that there are various active constituents with different polarities distributed in the plant extract that could inhibit the different stages of the inflammatory and nociception reaction.

In the writhing test, only the DCM and BuOH fractions at a dose of 100 mg/kg reduced the nociceptive response in mice. The hexane, EtOAc and BuOH fractions at a dose of at 100 mg/kg reduced the mice response to nociception induced by intraplantar formalin injection during the first phase of nociception (neurogenic phase). However, the EEMT previously tested at doses at 50, 100 and 200 mg/kg was not able to reduce the flinches during the first phase. The antinociceptive activity of the three fractions (H, E and B) in relation to the lack of activity of EEMT during the first phase may be due to the presence of the bioactive constituents at higher concentrations.

Furthermore, the separation of the compound antagonists by polarity could decrease the activity of the bioactive compounds. During the second phase (inflammatory phase), the four fractions (H, D, E and B) showed good antinociception that was similar to the antinociception of the standard drugs morphine and indomethacin. The edema was only reduced by the EtOAc fraction (100 mg/kg) to a degree similar to the reduction observed with indomethacin. The hexane, EtOAc and BuOH fractions acted similarly to opioids because they blocked the first and second phases induced by formalin; in contrast, the DCM fraction appeared to exert only peripheral activity on the inflammatory process.

The fractions also showed good activities on the inflammation or nociception provoked by Cg administration. Different doses need to be tested to assess the migration of neutrophils to the peritoneal cavity. The hexane fraction needed a higher dose (200 mg/kg) to show activity, whereas the EtOAc fraction required a lower dose (50 mg/kg). In the Von Frey test, the hexane, EtOAc and BuOH fractions reduced hypernociception in the mice. In the Evans blue test, the DCM fraction reduced the peritoneal exudate. Once again, we observed that one fraction had antinociceptive activity in a test in which EEMT was not effective. The higher concentration of the active compounds or the separation of antagonistic compounds could explain this effect. The reduction of the vascular permeability induced by Cg suggested an inhibition of the release of bradykinin, histamine and serotonin and the release of prostaglandins during a later stage of acute inflammation (3–4 h) by local cells [61].

The extract and fractions of *M*. *tenuiflora* bark probably act directly or indirectly by blocking the cascade of cytokines and the release of pro-inflammatory substances (bradykinin, histamine and serotonin) during the early phase of acute inflammation (1/2-1 h), culminating in the release of PG during the later stage of acute inflammation (3–4 h) by local cells. The effect could also be direct via the inhibition of a cyclooxygenase enzyme, such as indomethacin. These processes should be keys to the antinociceptive and anti-inflammatory activities of *M*. *tenuiflora*.

In an attempt to isolate the bioactive compound, the ethanolic extract of the bark and leaves of *M*. *tenuiflora* were tested in the writhing test (data not shown). The ethanolic extract of the *M*. *tenuiflora* bark showed higher antinociceptive activity than the leaves. However, the yield of the ethanolic extract of the leaves was greater than the bark extract. Sakuranetin was isolated from the leaves of *M*. *tenuiflora* to obtain better quantity, but sakuranetin was also present in the bark of this plant.

Pretreatment with the isolated sakuranetin at a dose of 200 mg/kg not only decreased the writhes induced by acetic acid in the peritoneum, but also reduced the neutrophil migration in mice induced by intraperitoneal injection of Cg, thereby demonstrating antinociceptive and anti-inflammatory activities. In contrast, the synthetized isosakuranetin at a dose of 200 mg/kg did not show significant antinociceptive and anti-inflammatory activities in the tests with mice. The differences in antinociceptive and anti-inflammatory actions between sakuranetin and the synthesized isosakuranetin may be explained by the fact that the product was synthesized as a racemic mixture. In contrast, sakuranetin had an enantiomeric excess of *S* because it was a natural compound biosynthesized in the presence of enantiomeric enzymes.

The antinociceptive and anti-inflammatory effects of sakuranetin could also contribute to this effect in the crude extract. Flavonoids as sakuranetin have been associated to anti-inflammatory and antioxidant activities. Toledo et al. evaluated the effects of sakuranetin treatment at experimental asthma model in mice, and demonstrated that sakuranetin attenuated lung inflammation and remodeling, probably by reducing the Th2 pro-inflammatory cytokines and oxidative stress [62]. Other possible mechanism of anti-inflammatory action of sakuranetin can be the control of NF-ĸB activation [62], the master regulators of pro-nociceptive factors, regulating the transcription of various genes, including cytokines (IL-1beta, IL-18) and iNOS [63]. The presence of hydroxyl group at C-7 and methoxyl group at C-5 at sakuranetin could be associated to the anti-inflammatory activity of this compound [64].

The crude extract showed activity at a lower concentration than sakuranetin, probably because there are other active compounds in EEMT or the effect was enhanced by the synergism that may occur between different substances in the extract. Groups treated with aqueous extract and fractions from *M*. *tenuiflora* showed a significant inhibition of cell migration (leukocyte) to the peritoneal cavity, similar inhibition obtained in our study, and also showed a reduction in the levels of IL-6, IL-12, and IL-1*β* in a antivenom *in vivo* test [65]. One hypothesis for this activity is the inhibition of the production of leukotrienes and prostaglandin E2 by inhibiting the enzyme phospholipase A2 and COX2, respectively, because the presence of saponins in this species [65]. The anti-inflammatory activity of saponins and flavonoids may be mediated by inhibiting the activation of nuclear factor-kB, thus resulting in decreased expression of NF-kB-regulated proteins [66, 67].

In summary, the present study provided evidence that the EEMT crude extract, its fractions and the isolated compound sakuranetin showed significant anti-inflammatory and antinociceptive activities when administered subcutaneously to mice. Despite the great number of available anti-inflammatory and analgesic drugs, their side effects and the ineffectiveness of some drugs in some conditions require the continuous search for new drugs. These data support the development of additional studies to identify the other active constituents of the *M*. *tenuiflora* bark extract and to elucidate their mechanism of action for the development of novel phytomedicines.

## Supporting Information

S1 Fig *Mimosa tenuiflora* (Willd.) Poiret (COSTA, 2011). (TIF)

S2 Fig Synthesis of isosakuranetin. (TIF)

S3 Fig Chemical structure and ^1^H NMR spectral data of sakuranetin, 1 [500 MHz, CDCl_3_, δ (ppm)]. (TIF)

S4 Fig MS spectral data of sakuranetin (1). (TIF)

S5 Fig MS spectral data of isosakuranetin (2). (TIF)

S6 Fig Chemical structure and ^1^H NMR spectral data of genkwanin (3) [300 MHz, DMSO-d^6^, δ (ppm)]. (TIF)

S7 Fig Chemical structure and ^1^H NMR spectral data of sorbifolin (4) [300 MHz, DMSO-d^6^, δ (ppm)]. (TIF)

S8 Fig Chemical structure and ^1^H NMR spectral data of 5,4’-di-hydroxy-7,8-dimethoxyflavone (5) [300 MHz, CDCl_3_, δ (ppm)]. (TIF)

S9 Fig Chemical structure and ^1^H NMR spectral data of 5,7,4’-trihydroxy-3-methoxyflavone (6) [300 MHz, CD_3_OD, δ (ppm)]. (TIF)

S10 Fig Chemical structure and ^1^H NMR spectral data of 5,7,4’-trihydroxy-6-methoxyflavonol (7) [300 MHz, CO(CD_3_)_2_, δ (ppm)]. (TIF)

S11 Fig Chemical structure and ^1^H NMR spectral data of 5,6-dihydroxy-7,4’-dimethoxyflavonol (8) [300 MHz, CO(CD_3_)_2_, δ (ppm)]. (TIF)

S12 Fig Chemical structure and ^1^H NMR spectral data of 5-hydroxy-7,8,4’-trimethoxyflavonol (9) [300 MHz, CDCl_3_, δ (ppm)]. (TIF)

S13 Fig Effect of ethanol extract of *M*. *tenuiflora* bark and leaf in writhing test induced by acetic acid (0.6%) of mice pretreated with 200 mg/kg. VH are mice treated with vehicle (negative control). Results are presented as means ± S.D. of writhing in mice (n = 8). Statistical significance was calculated by ANOVA followed by Bonferroni's test. *P < 0.05 compared to the vehicle-treated group.
(TIF)
