# Supplementary figures and images for: Antinoceptive and Anti-inflammatory Activities of the Ethanolic Extract, Fractions and Flavones Isolated from Mimosa tenuiflora (Willd.) Poir (Leguminosae)
Source: PLoS One. 2016 Mar 8;11(3):e0150839. doi: 10.1371/journal.pone.0150839 (PMC4783012; doi:10.1371/journal.pone.0150839)

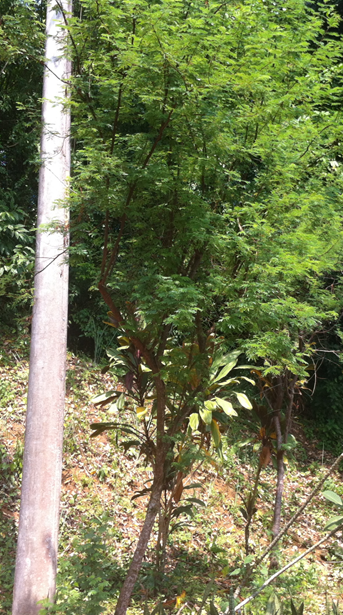

Supplement: S1 Fig — (TIF) [file pone.0150839.s001.tif]

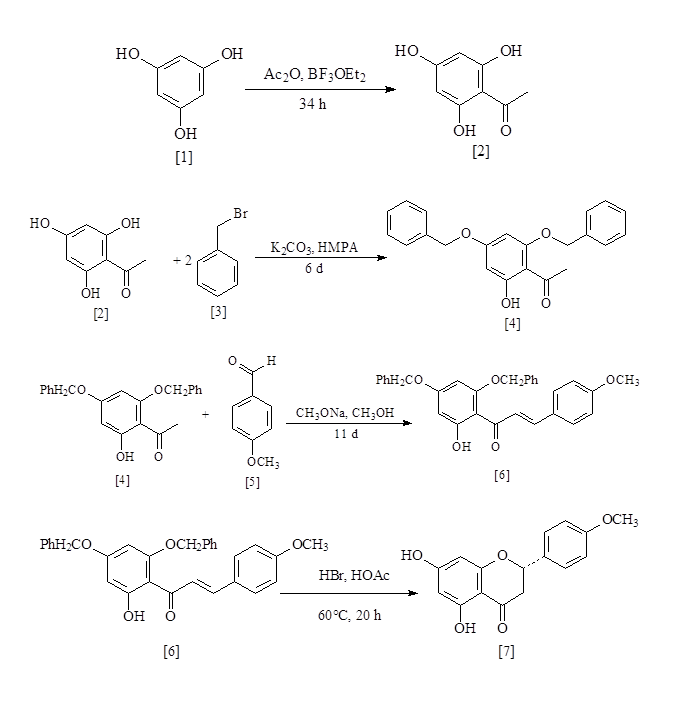

Supplement: S2 Fig — (TIF) [file pone.0150839.s002.tif]

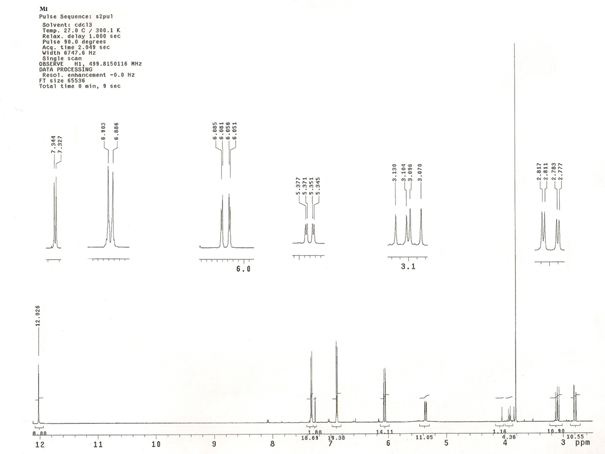

Supplement: S3 Fig — (TIF) [file pone.0150839.s003.tif]

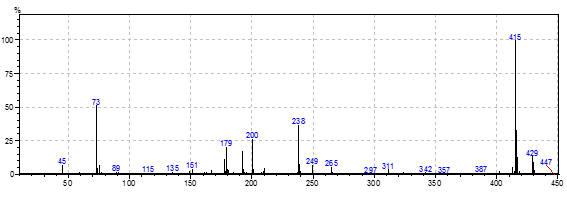

Supplement: S4 Fig — (TIF) [file pone.0150839.s004.tif]

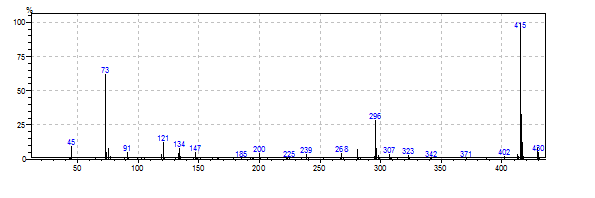

Supplement: S5 Fig — (TIF) [file pone.0150839.s005.tif]

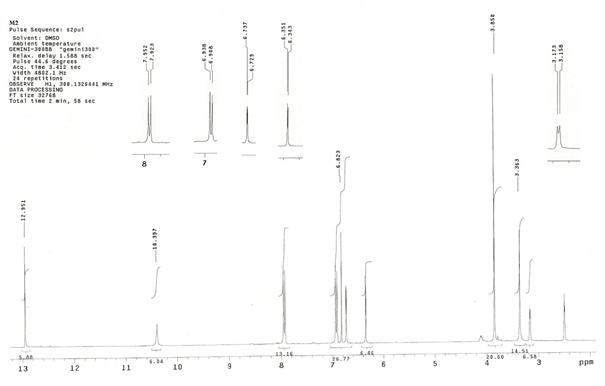

Supplement: S6 Fig — (TIF) [file pone.0150839.s006.tif]

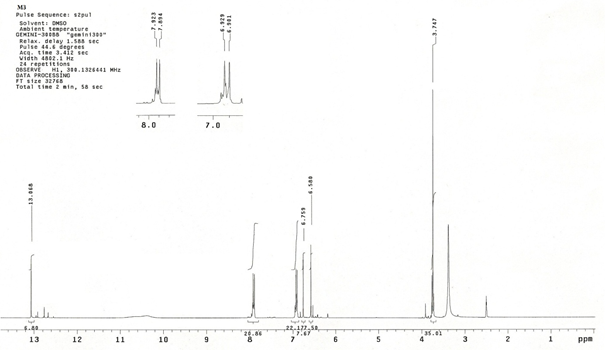

Supplement: S7 Fig — (TIF) [file pone.0150839.s007.tif]

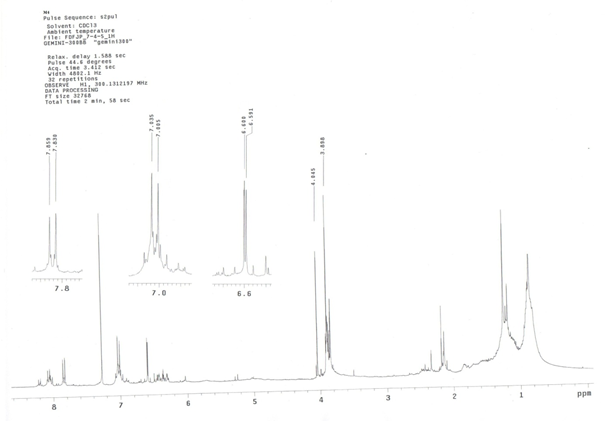

Supplement: S8 Fig — (TIF) [file pone.0150839.s008.tif]

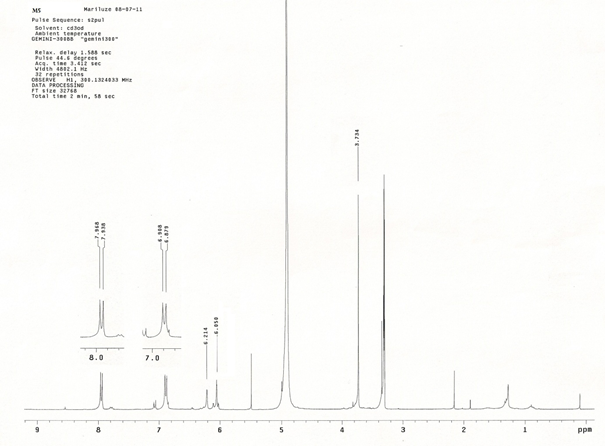

Supplement: S9 Fig — (TIF) [file pone.0150839.s009.tif]

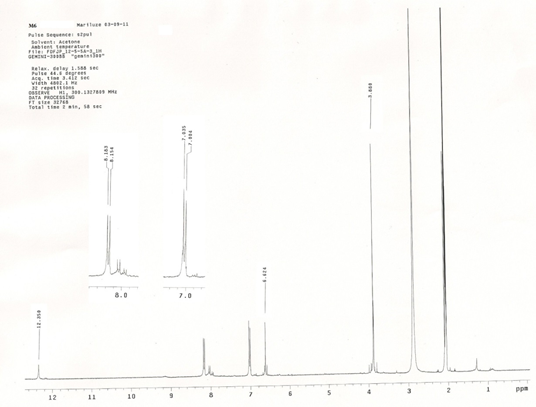

Supplement: S10 Fig — (TIF) [file pone.0150839.s010.tif]

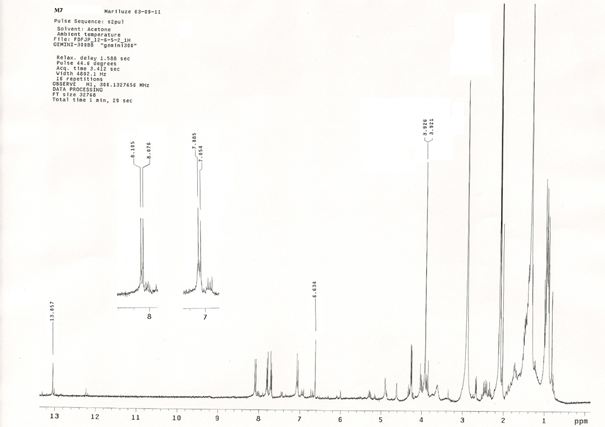

Supplement: S11 Fig — (TIF) [file pone.0150839.s011.tif]

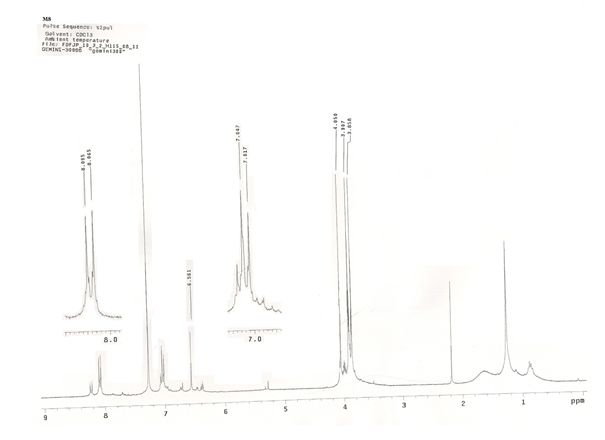

Supplement: S12 Fig — (TIF) [file pone.0150839.s012.tif]

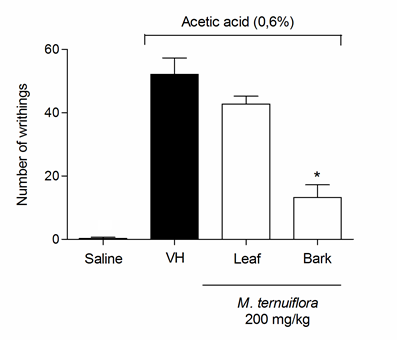

Supplement: S13 Fig — VH are mice treated with vehicle (negative control). Results are presented as means ± S.D. of writhing in mice (n = 8). Statistical significance was calculated by ANOVA followed by Bonferroni's test. *P < 0.05 compared to the vehicle-treated group. (TIF) [file pone.0150839.s013.tif]
